# Spin-Restricted Descriptions of Singlet Oxygen Reactions from XMS-CASPT2 Benchmarks

**DOI:** 10.1021/acs.jpca.4c00744

**Published:** 2024-05-13

**Authors:** Max Winslow, Alexander Hazelby, David Robinson

**Affiliations:** Department of Chemistry and Forensics, School of Science and Technology, Nottingham Trent University, Clifton Lane, Nottingham NG11 8NS, United Kingdom

## Abstract

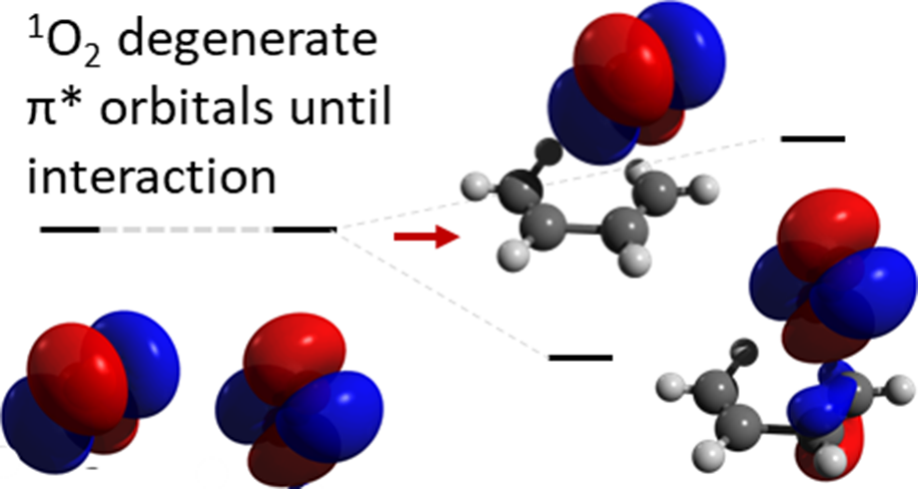

Reactions of singlet oxygen are numerous, some of which are desired but many are unwanted. Therefore, the ability to correctly predict and interpret this reactivity for complex molecular systems is essential to our understanding of singlet oxygen reactions. DFT is widely used for predicting many reactions but is not suited to degenerate electronic structures; application to isolated singlet oxygen often uses the spin-unrestricted formalism, which results in severe spin contamination. In this work, we demonstrate that spin-restricted DFT can correctly describe the reaction pathway for four prototypical singlet oxygen reactions. By careful benchmarking with XMS-CASPT2, we show that, from the first transition state onward, the degeneracy of the ^1^Δ_g_ state is broken due to differing interactions of the (degenerate) π* orbitals with the organic substrate; this result is well replicated with DFT. These findings demonstrate the utility of using spin-restricted DFT to explore reactions, opening the way to confidently use this computationally efficient method for molecular systems of medium to large organic molecules.

## 1 Introduction

The highly reactive first singlet excited state of molecular oxygen (^1^Δ_g_, often referred to as “singlet oxygen”) is often produced by photosensitization via the type II process of reactive oxygen species. In this process, intersystem crossing (ISC) occurs from the triplet state (ground electronic state) to the ^1^Δ_g_ singlet state. Reactions of singlet oxygen are found in many areas of chemistry, from desired reactions (e.g., wastewater treatment,^1,2^ organic synthesis reactions,^3^ and photodynamic therapy^4−6^) to situations where singlet oxygen causes much damage (e.g., reactions with DNA bases,^7−9^ vitamins, and other foodstuffs;^10−15^ oxidative stability of electrolytes in Li-ion batteries,^16^ among many others). There are many experimental techniques to detect the presence of singlet oxygen in chemical systems and thus aid our understanding of the situations and environments in which singlet oxygen production (and quenching^17^) is possible. This can become complicated, as some molecules which act as singlet oxygen quenchers can become photosensitizers at lower concentrations.^11^ Understanding the reactivity of singlet oxygen is therefore of critical importance to modulating reactions, desired or otherwise.

There has been much discussion regarding the application of single-reference methods, particularly density functional theory (DFT), to reactions involving singlet oxygen, due to the doubly degenerate nature of the ^1^Δ_g_ electronic state of O_2_.^18−23^ Ponra et al.^23^ investigated the application of the multiplet sum method (MSM)^24,25^ DFT approach to correctly describe the potential energy curves for the lowest three electronic states of O_2_ (^3^Σ_g_^–^, a^1^Δ_g_, and b^1^Σ_g_^+^). They found that simply fixing the spin multiplicity with standard DFT was not sufficient to describe the potential energy curve for the ^1^Δ_g_ state. Garavelli et al.^20^ used a spin-projection method^21^ to try and improve the excitation energy from the triplet ground state to the ^1^Δ_g_ state. Lee and Head-Gordon^26^ tested variants of regularized orbital optimized Mo̷ller-Plesset perturbation theory (OOMP2) for application to singlet oxygen reactions. There are numerous examples in which the spin multiplicity is fixed to unity, and the spin-unrestricted approach is employed (with or without spin-projection corrections; see, for example, refs (18,27)).

In this article, we investigate and benchmark the use of spin-restricted Kohn–Sham (RKS) DFT for singlet oxygen reactions and compare several different key properties with those calculated at the high-level (extended) multistate multireference perturbation theory, (X)MS-CASPT2. We postulate that, although such an approach cannot correctly describe the doubly degenerate nature of the ^1^Δ_g_ state of O_2_ (and thus the potential energy curve), the double-degeneracy is lifted upon interaction with an organic reactant molecule and disappears as the reaction proceeds, thus a spin-restricted formalism could be a highly desirable approach due to its computational efficiency and simplicity.

## 2 Electronic Structure of Molecular Oxygen

**Figure 1:**
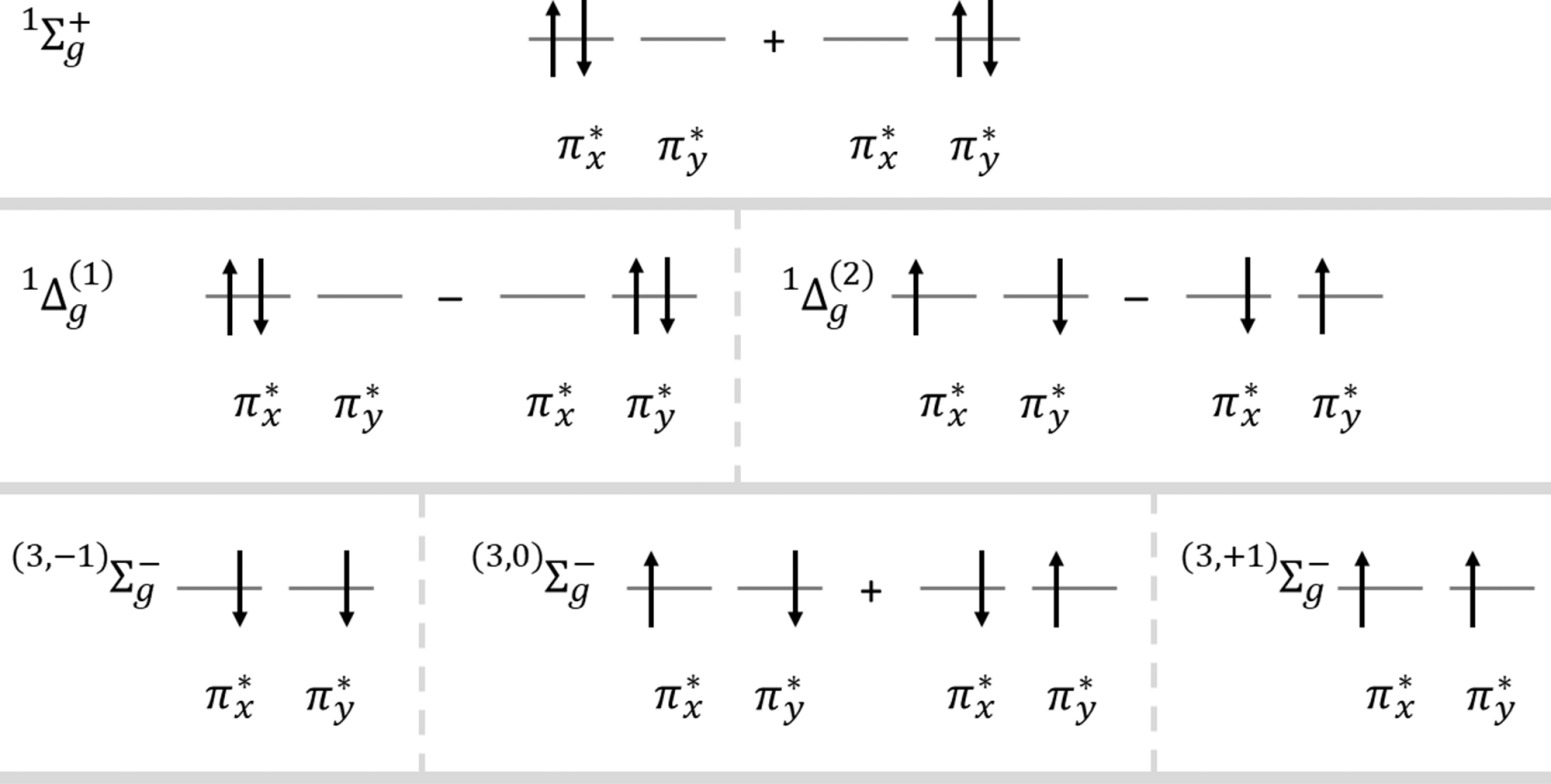
Schematic representation of the wave functions for the lowest triplet and two lowest singlet electronic states of O_2_ (only the degenerate π* orbitals shown for clarity).

Before considering the interaction of singlet oxygen with other molecules, it is worth considering in detail the electronic structure of the lowest electronic states of isolated O_2_. Following the notation of Ponra et al.,^23^ the ground state of O_2_ is a triplet with the following configurations (within the symmetry adapted linear configurations of the D_∞h_ point group; see Figure 1 for a visual representation):
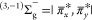
1a
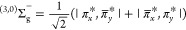
1b
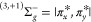
1c

The first singlet excited state, ^1^Δ_g_, has the following two degenerate configurations:
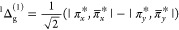
2a
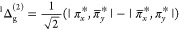
2b

Finally, the second singlet excited state, ^1^Σ_g_^+^, is
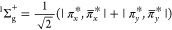
3We will use only the real-valued orbitals in this work; Ponra et al. give a good discussion on the equivalence of the wave functions written with either real-valued or complex-valued molecular orbitals.^23^ It is clear from the form of the wave functions above that a single-determinant method (including DFT) is not sufficient to correctly describe the electronic states of interest of the isolated O_2_ molecule. However, as the reactivity of ^1^O_2_ necessitates differing overlap of the degenerate π* orbitals with the substrate’s orbitals, then the degeneracy must break and the symmetry of the reacting singlet oxygen electronic state will no longer be degenerate.

## 3 Computational Details

**Figure 2:**
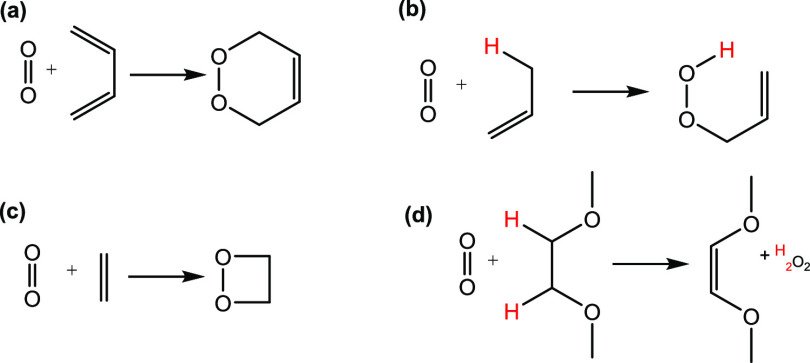
Common reaction motifs of singlet oxygen considered in this study: (a) 1,4-cycloaddition,^42^ (b) the “ene” reaction, (c) 1,2-cycloaddition, and (d) a recently proposed reaction in which two hydrogen atoms are abstracted at the same time.^43^ Where hydrogen atom transfer occurs (reactions (b) and (d)), only that hydrogen is explicitly shown.

**Figure 3:**
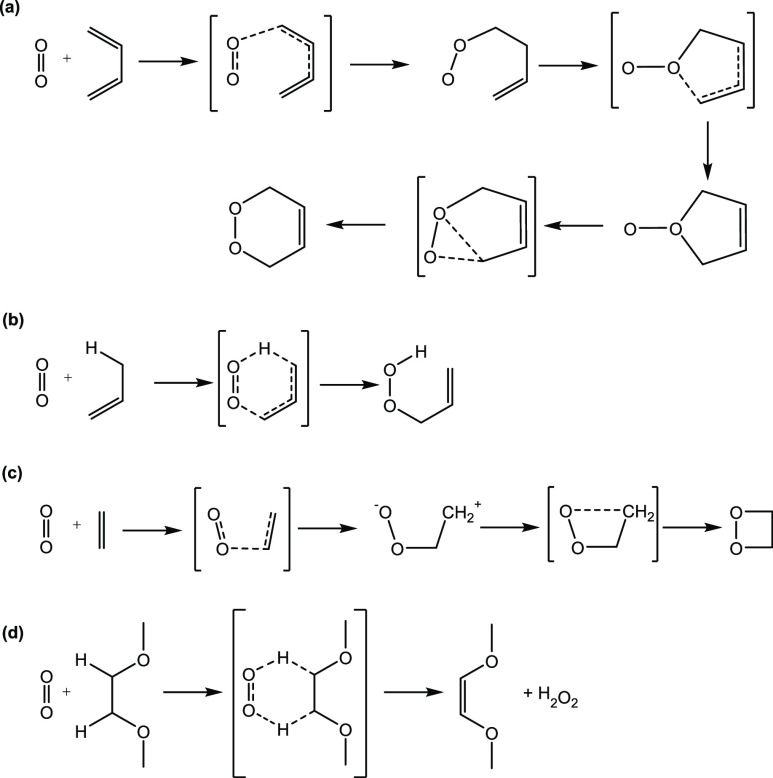
Predicted mechanisms for each of reactions (a–d) from Figure 1 using B3LYP/6-31G(d). Transition state structures are shown in square parentheses.

In this work, we considered four common (nonradical) reaction motifs of singlet oxygen (Figure 2). Geometries of the reactants, intermediates, products, and transition states were determined using the B3LYP,^28^ M11^29^ and ωB97X-D3^30^ functionals and 6-31G(d),^31^ and Def2-TZVPP basis sets.^32^ The nature of the stationary points was confirmed by harmonic vibrational frequency calculations, with all minima having zero imaginary frequencies and all transition states having a single imaginary frequency. Reaction path calculations^33^ confirmed that the transition states connected the minima shown in Figure 3.

Potential energy surfaces (PES) around the first transition state for each reaction were determined using the geometries from the B3LYP, M11, and ωB97X-D3 minimum energy reaction path calculations using the 6-31G(d) basis set. The complete active space self-consistent field (CASSCF) wave function included the lowest two singlet states using state-averaging (for the singlet calculations) and a single triplet state as a separate calculation. Full details of the active space selection for each reaction are given in the Supporting Information. The XMS-CASPT2^34^ calculations used a real shift of 0.2 au, while the single-state single-reference (SS-SR) contraction scheme was used. The 6-31G(d) basis set was used, along with the density fitting approximation for the two-electron integrals, employing the TZVPP-JKFIT density fitting basis set.^35^ All XMS-CASPT2 calculations were performed with the BAGEL software suite.^36,37^ The CASSCF calculations were repeated with Molpro^38^ to obtain energies of the canonical orbitals. Equivalent DFT curves were calculated using time-dependent DFT (TDDFT), within the Tamm-Dancoff approximation (TDA),^39^ using the B3LYP, M11, and ωB97X-D3 functionals. The TDDFT/TDA approach was necessary, as the reference Kohn–Sham determinant has a triplet instability by design (the triplet state is the ground state), which leads to a negative eigenvalue in the orbital Hessian.^40^ As this is not used within TDA, diagonalization of the **A** matrix is possible.

Reaction enthalpy profiles were determined using XMS-CASPT2, B3LYP, M11, and ωB97X-D3 functionals, with the 6-31G(d), def2-TZVPP, and def2-QZVPP^32,35^ basis sets using single-point energy calculations based on the B3LYP/6-31G(d) optimized geometries. The geometries of all the points were also optimized using M11 and ωB97X-D3 with the 6-31G(d) basis set to determine any differences in stationary points obtained with different functionals. All DFT calculations were performed with Q-Chem 5.4.^41^

## 4 Results and Discussion

### 4.1 Results

The mechanisms determined by the DFT calculations (except M11 for reaction (a); see further discussion below) are shown in Figure 3, with energies discussed later. The reaction mechanism for reaction (a) closely follows one of the pathways identified by Bobrowski et al.,^42^ in which they determined geometries at the CASSCF level, with energetic corrections using MCQDPT2. The mechanism for reaction (b) resembles one of the pathways determined by Maranzana et al.,^44^ and the mechanism for reaction (c) resembles one of those identified by Tonachini et al.^45^ The mechanism for reaction (d) is based on that seen for similar molecules proposed by Freiberg et al.^43^

**Figure 4:**
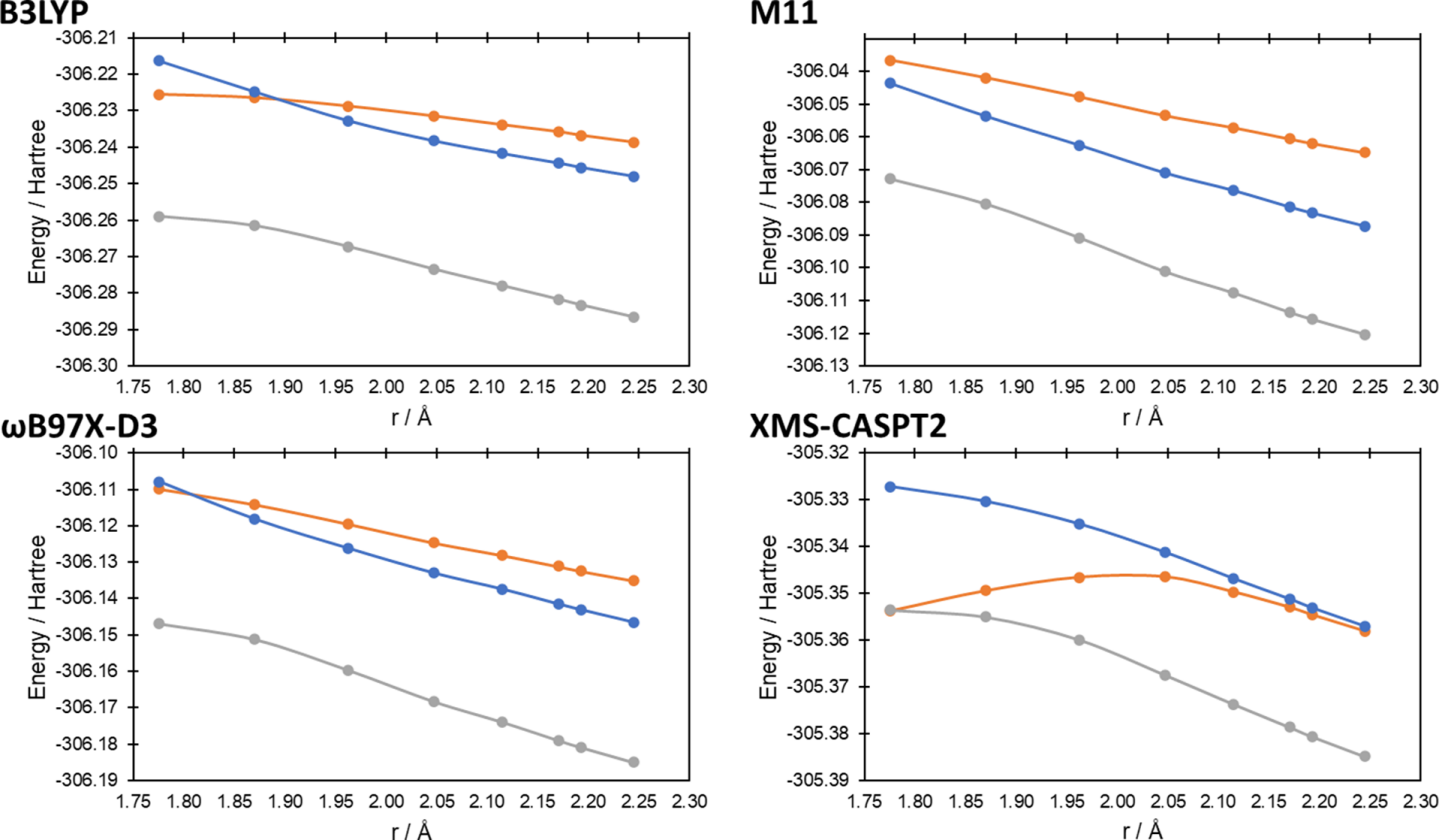
Potential energy curves for the lowest triplet and two lowest singlet energy states at the first transition state geometry (r_C–O_ = 1.78 Å; data point on the left) and along the intrinsic reaction coordinate calculated at the B3LYP/6-31G(d) level for reaction (a). Distance on the *x*-axis corresponds to the C–O bond distance as the bond forms (see Figure 2a) along the reaction coordinate. Triplet energies are the gray lines, while the singlet energies are the orange and blue lines.

**Figure 5:**
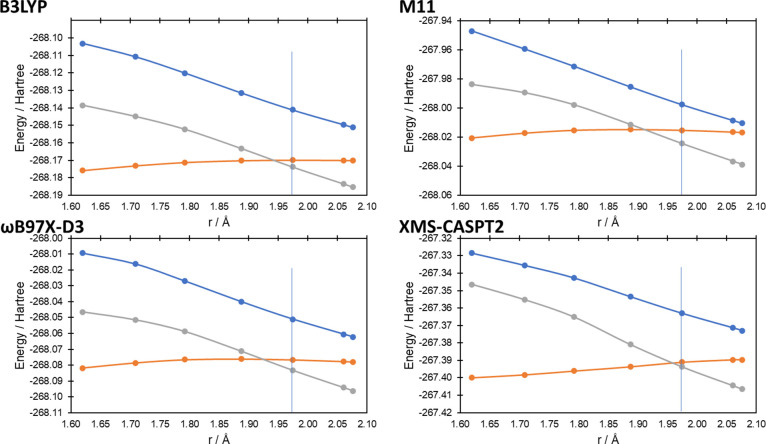
Potential energy curves for the lowest triplet and two lowest singlet energy states at the first transition state geometry (r_C–O_ = 1.97 Å; data point highlighted with a vertical line) and along the intrinsic reaction coordinate calculated at the B3LYP/6-31G(d) level for reaction (b). Distance on the *x*-axis corresponds to the C–O bond distance as the bond forms (see Figure 2b) along the reaction coordinate. Triplet energies are the gray lines, while the singlet energies are the orange and blue lines.

**Figure 6:**
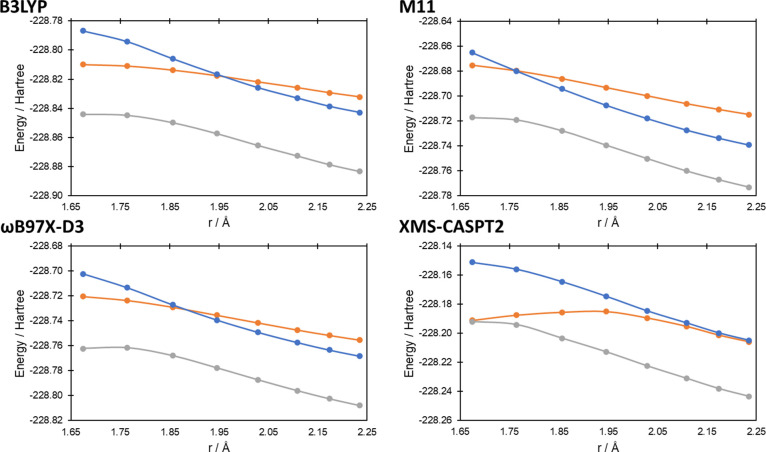
Potential energy curves for the lowest triplet and two lowest singlet energy states at the first transition state geometry (r_C–O_ = 1.67 Å; data point on the left) and along the intrinsic reaction coordinate calculated at the B3LYP/6-31G(d) level for reaction (c). Distance on the *x*-axis corresponds to the C–O bond distance as the bond forms (see Figure 2c) along the reaction coordinate. Triplet energies are the gray lines, while the singlet energies are the orange and blue lines.

**Figure 7:**
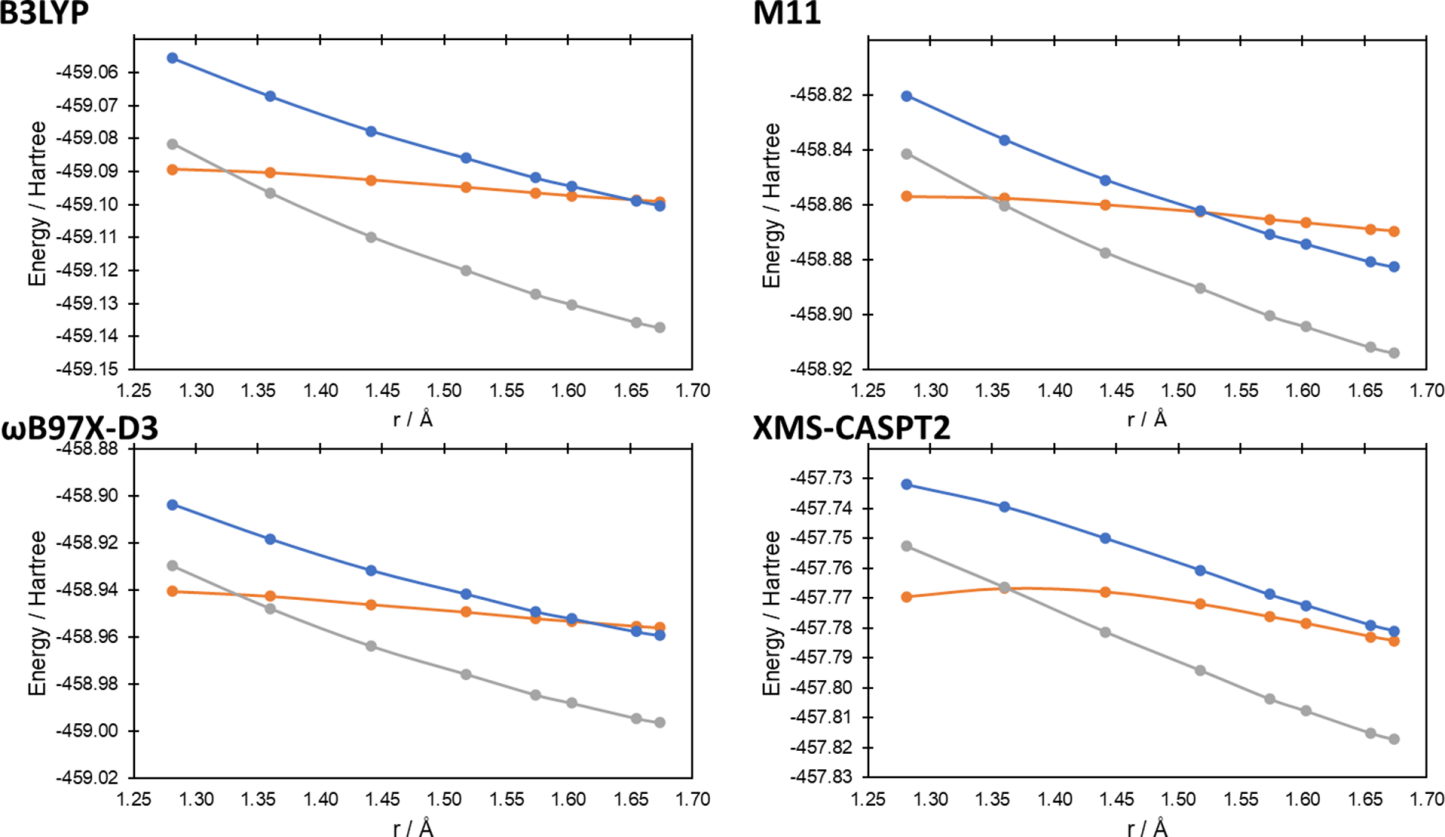
Potential energy curves for the lowest triplet and two lowest singlet energy states at the first transition state geometry (r_O–H_ = 1.28 Å; data point on the left) and along the intrinsic reaction coordinate calculated at the B3LYP/6-31G(d) level for reaction (d). Distance on the *x*-axis corresponds to the O–H bond distance as the bond forms (see Figure 2d) along the reaction coordinate. Triplet energies are the gray lines, while the singlet energies are the orange and blue lines.

In a recent paper, Mullinax et al.^16^ postulated that for reactions of organic molecules with singlet oxygen leading to products with a singlet electronic state, the singlet and triplet PES must cross along the reaction coordinate to avoid collisional deactivation of singlet oxygen. While the agreement between their CASPT2 and M11 calculations was qualitatively different, they both predicted that the lowest singlet and triplet states were within 10 kcal mol^–1^ of each other at the transition state geometries. In Figures 4–7 we present the potential energy curves for the lowest singlet and triplet energies along the reaction coordinate of reactions (a–d), respectively, where the reaction coordinate is the reaction pathway determined by B3LYP/6-31G(d); equivalent potential energy curves using the reaction pathways using M11/6-31G(d) and ωB97X-D3/6-31G(d) can be found in Figures S1–S4 and Figures S5–S8, respectively, in the Supporting Information. In all cases at the XMS-CASPT2 level, the influence of the ^1^Δ_g_ state of O_2_ can be seen at longer bond lengths (i.e., before the O = O bond breaks), with the two lowest singlet state energies being degenerate (or near-degenerate in the case of reaction (c), Figure 5). In all cases, as *r* approaches the transition state, the degeneracy of the two singlet states breaks, while the lowest singlet state becomes degenerate with the triplet state (reactions (a–c)) and nearly degenerate for reaction (d). For the 1,4-cycloaddition, ene reaction and 1,2-cycloaddition reactions (reactions (a–c), Figures 4–6, respectively), the lowest singlet and triplet states’ curves cross at the transition state (singlet–triplet gaps of 0.1, −1.4, and −0.6 kcal mol^–1^ for reactions (a–c), respectively; a negative value indicates the T_1_ state being lowest in energy), while for reaction (d) (Figure 7), it is clear that the crossing is very close to the transition state (2.0 kcal mol^–1^ at a O–H bond distance 0.035 Å longer than the transition state).

**Figure 8:**
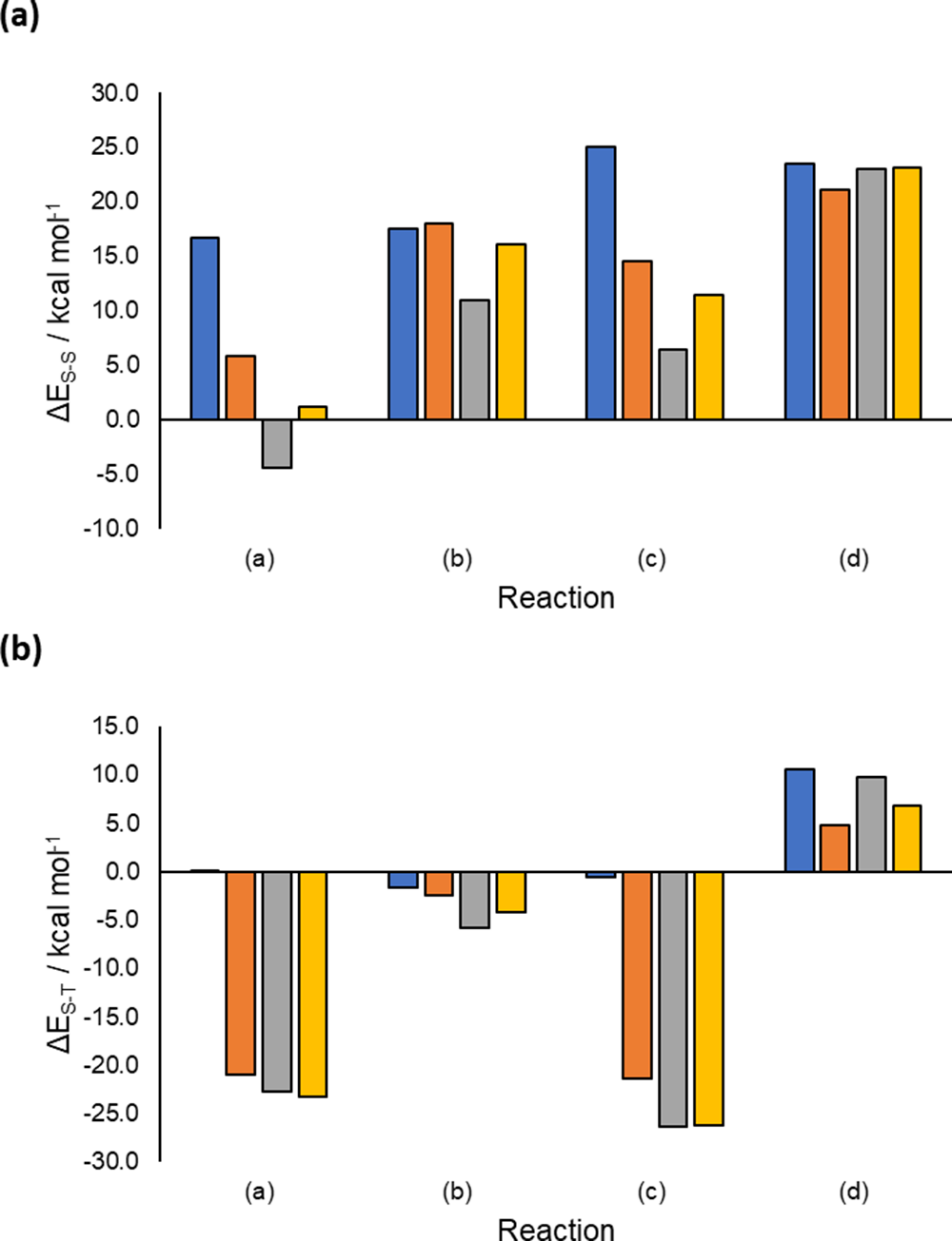
(a) Singlet–singlet energy gaps at the transition state geometry for XMS-CASPT2 (blue), B3LYP (orange), M11 (gray), and ωB97X-D3 (yellow). Positive values for the DFT-based methods indicate the SCF solution is lower in energy than the TDDFT/TDA first singlet excited state. (b) Singlet–triplet energy gaps at the transition state geometry for XMS-CASPT2 (blue), B3LYP (orange), M11 (gray), and ωB97X-D3 (yellow). Negative values indicate that the triplet state is the ground state. All calculations use the 6-31G(d) basis set.

We next consider the potential energy curves generated by spin-restricted DFT/TDDFT/TDA (Figures 4–7). For reaction (a), none of the DFT methods qualitatively matches the XMS-CASPT2 curves, with B3LYP and ωB97X-D3 both exhibiting (near) degeneracy of the two singlet states along the whole potential energy curve, while M11 shows degeneracy at the transition state, which breaks further from the transition state geometry, a reversal of the trend seen for XMS-CASPT2. For M11, the lowest singlet state from the TDDFT/TDA calculations is lower than the SCF solution, indicating an internal instability. Further investigation shows that this leads to the undesired spin-unrestricted (UKS) solution. Optimization of the transition state geometry with spin-restricted M11 leads to a concerted structure (similar to transition state 2, reaction (a), Figure 3); the three step reaction scheme is not found at the RKS M11 level. None of the methods show degeneracy of the singlet and triplet states at the transition state of reaction (a) (Figure 8). For reaction (b), B3LYP matches the XMS-CASPT2 curves both qualitatively and quantitatively for both the singlet–singlet and singlet–triplet gaps along the curve (Figures 4 and 8). The M11 and ωB97X-D3 curves show good agreement near the transition state, but the degeneracy of the singlet states further from the transition state is quantitatively incorrect, although the maximum nondegeneracy is 11.3 kcal mol^–1^ for M11 at the furthest point from the transition state (Figure 8). For reaction (c), B3LYP exhibits the best correlation with the XMS-CASPT2 curve, at least qualitatively correct for the breaking of the degenerate singlet state approaching the transition state. In all cases for the DFT-based approaches, the difference between the singlet and triplet states at the transition state is >11 kcal mol^–1^, compared to 0.6 kcal mol^–1^ for XMS-CASPT2 (Figure 8). For reaction (d), all of the functionals are qualitatively correct, with reasonable quantitative accuracy. With the exception of reaction (a) for M11, the potential energy curves calculated using the M11 and ωB97X-D3 reaction pathways (Figures S2–S8) qualitatively match Figures 1, 4 above for the DFT methods. For reaction (b), the XMS-CASPT2 curves look qualitatively different at C–O bond lengths shorter than the transition state (i.e., as the product is formed) but otherwise match the discussion given for the B3LYP reaction pathway above.

**Figure 9:**
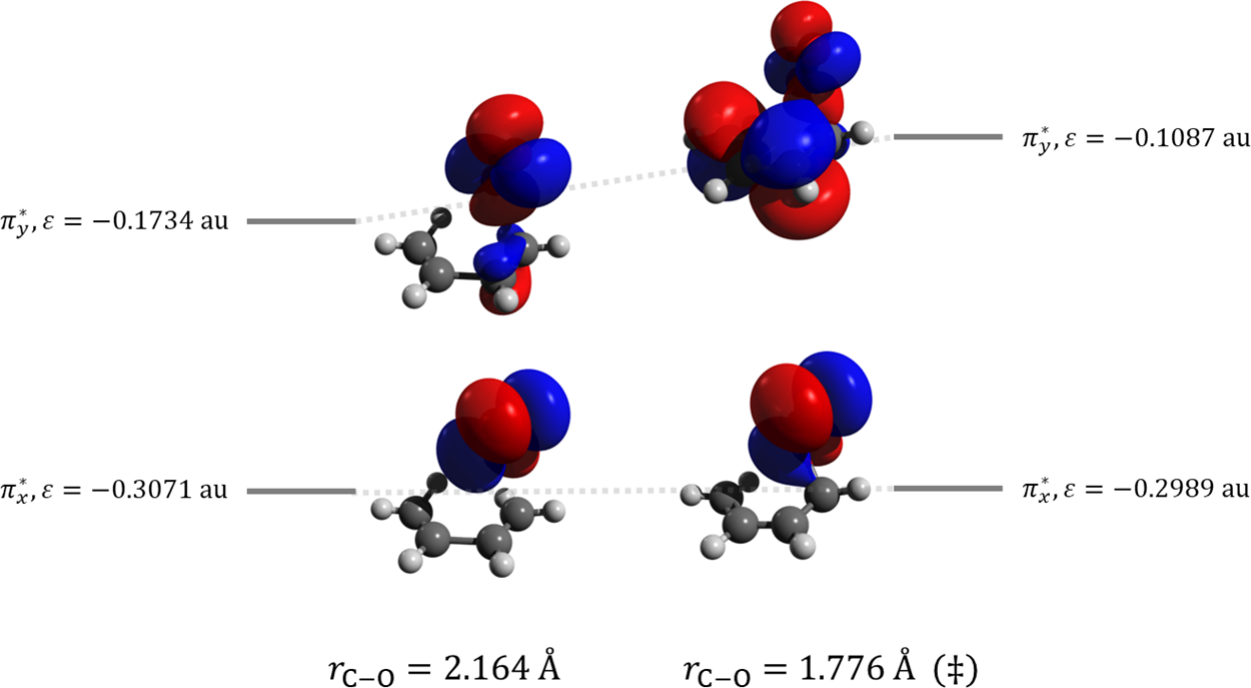
Schematic of the two π* orbitals of O_2_ interacting with butadiene a short distance from the transition state (left) and at the transition state (right) for reaction (a). Orbitals come from the CASSCF/6-31G(d) calculations.

**Figure 10:**
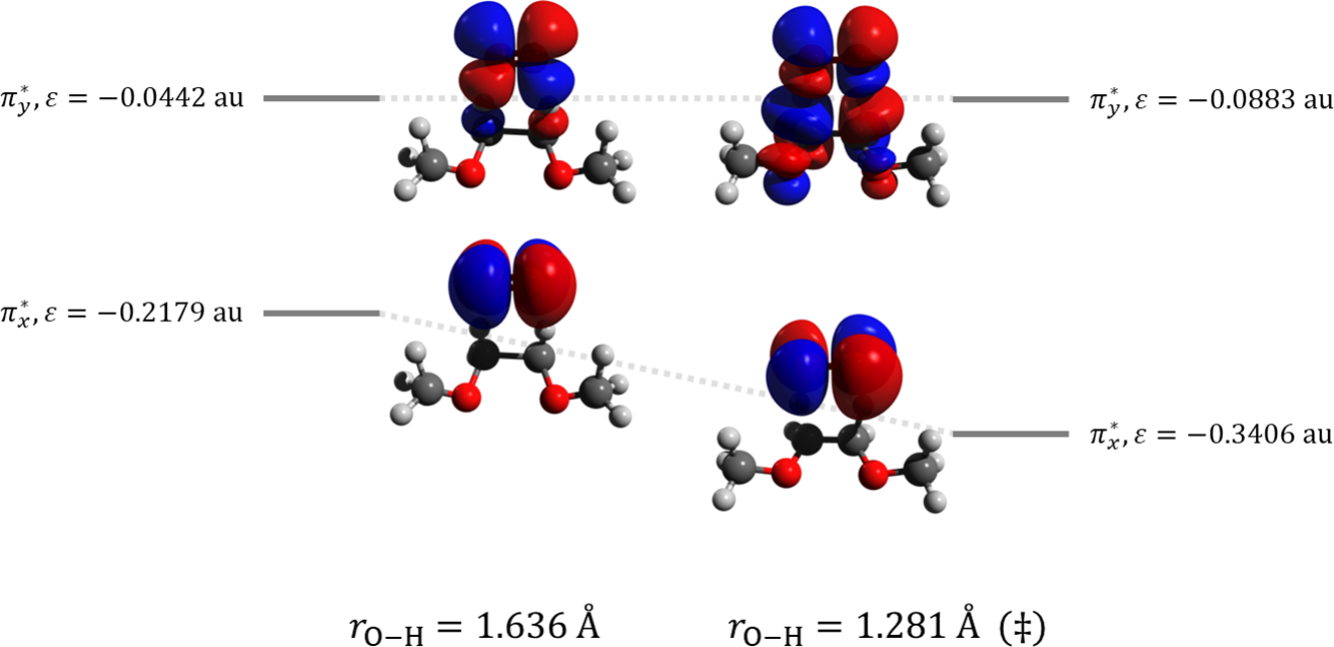
Schematic of the two π* orbitals of O_2_ interacting with 1,2-dimethoxyethane a short distance from the transition state (left) and at the transition state (right) for reaction (d). Orbitals come from CASSCF/6-31G(d) calculations.

Shown in Figure 9 are the two CASSCF π* orbitals that are degenerate in isolated O_2_, interacting with butadiene a short distance from the transition state and at the transition state for reaction (a). It is clear that the two orbitals are not degenerate at either point, with the higher energy orbital interacting with a π* orbital of butadiene and moving higher in energy at the transition state. A similar pattern is seen for reactions (b,c) (Figures S9 and S10), where destabilization of the higher energy π* orbital occurs. For reaction (d), a different phenomenon is observed, where the lower energy π* orbital is stabilized at the transition state (Figure 10). In all cases, the interaction of ^1^O_2_ with the organic substrate leads to the degeneracy of the π* orbitals of O_2_ being lifted.

A similar analysis of the two π* orbitals for the DFT methods is given in Figures S11–S14. It is worth noting that Kohn–Sham and wave function-based orbitals cannot be directly compared, but for hybrid methods, the KS orbitals usually closely resemble those derived from wave function theory. For reaction (a), the DFT KS orbitals qualitatively match the CASSCF orbitals away from the transition state, although at the transition state, the lower energy orbital (π_x_^*^) shows more overlap with the butadiene orbitals for the KS orbitals than observed for the CASSCF orbitals (Figure S11). The π_y_^*^ orbital is destabilized at the transition state (relative to a distance further from the transition state) at the CASSCF level, with similar qualitative results for B3LYP and ωB97X-D3, while M11 exhibits destabilization of the π_x_^*^ orbital; however, in all cases, the two π* orbitals are nondegenerate. For reaction (b) (Figure S12), both B3LYP and M11 qualitatively match CASSCF, both orbital shapes and energies. The ωB97X-D3 orbital shapes are also similar, although both orbitals show destabilization at the transition state, rather than stabilization (π_x_^*^) and destabilization (π_y_^*^) seen for CASSCF. For reaction (c), B3LYP qualitatively matches the trends seen for CASSCF (Figure S13). The π_x_^*^ orbitals from all DFT methods show much more significant overlap with the ethene π orbital than seen for CASSCF. Further investigation revealed that Hartree–Fock (HF) orbitals look similar to the CASSCF orbitals, while BLYP orbitals (a pure DFT method) were considerably different. The hybrid functionals used here are intermediate between the HF and BLYP orbitals. For reaction (d), each of the DFT methods shows good qualitative agreement with CASSCF.

**Figure 11:**
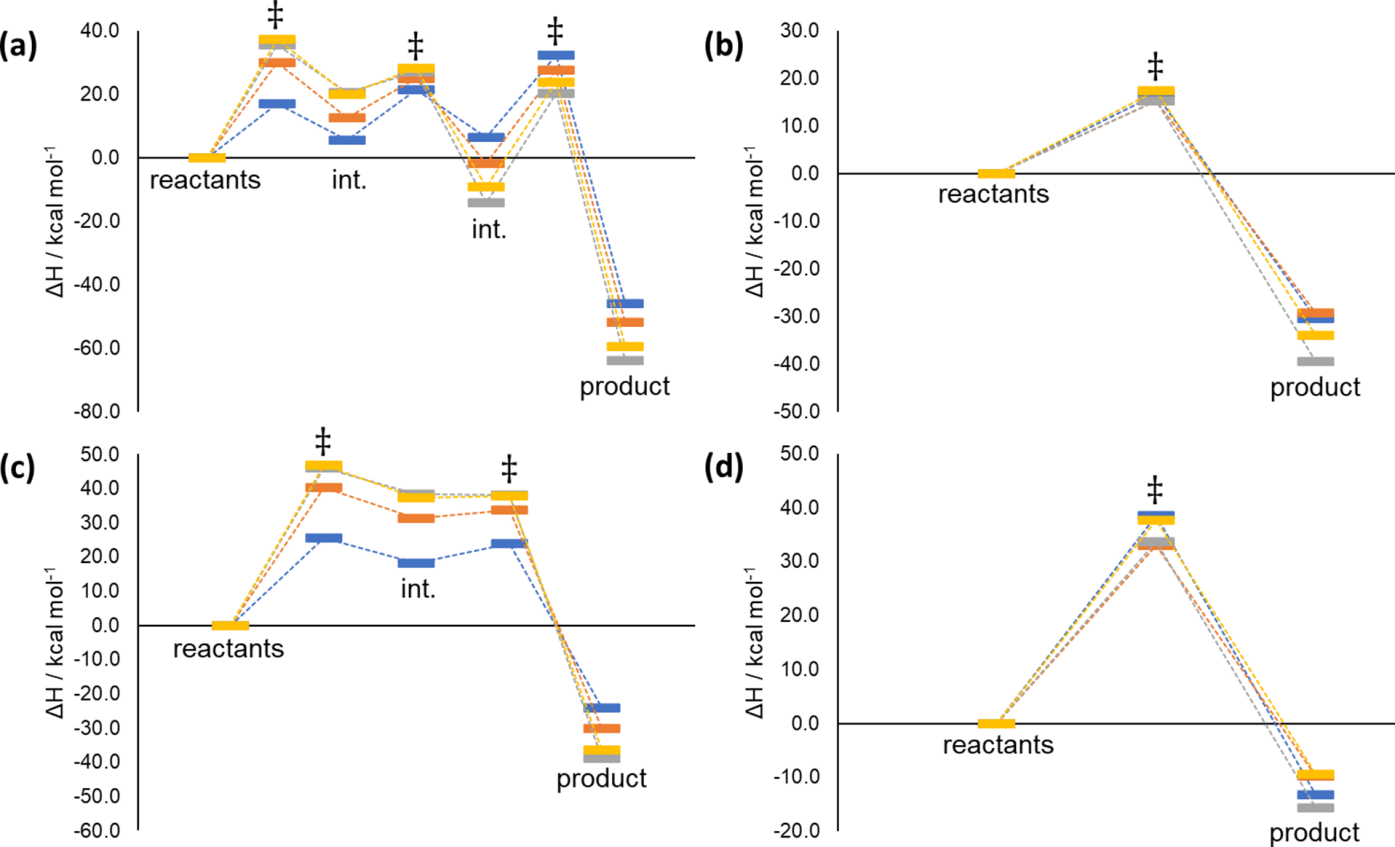
Reaction enthalpy profiles for reactions (a–d) using the 6-31G(d) basis set. Absolute value of the singlet oxygen energy for the DFT methods is taken as the sum of the triplet ground state energy plus the triplet–singlet gap taken from the XMS-CASPT2 calculations. Key: XMS-CASPT2 (blue), B3LYP (orange), M11 (gray), and ωB97X-D3 (yellow).

Given in Figure 11 are the reaction enthalpy profiles for each of reactions (a–d), where the singlet oxygen total energy for the reactants for the DFT methods is given as
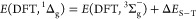
4where Δ*E*_S–T_ is the energy gap between the ^3^Σ_g_^–^ and a^1^Δ_g_ electronic states. This correction is used to account for the incorrect description of isolated singlet oxygen with a single-reference method. There are many possible ways to estimate this metric; we have used the MSM-DFT scheme proposed by Ponra et al.,^23^ in which:
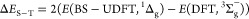
5

In eq 5, *E*(BS–UDFT, ^1^Δ_g_) corresponds to the energy of the ^1^Δ_g_ state of the isolated O_2_ molecule calculated broken spin symmetry DFT, which typically has an ⟨*S*^2^⟩ value of approximately 1.0. In general, the Δ*H*^‡^ values for the transition states are higher with DFT than XMS-CASPT2, while the Δ_r_*H* values are typically more negative for the DFT methods than seen for XMS-CASPT2 (see Table 1). B3LYP shows the best agreement overall, especially for the mean signed deviations for the transition state barrier heights. Given in Figures S15 and S16 are the reaction enthalpy profiles calculated with the larger basis sets, def2-TZVPP and def2-QZVPP, respectively. Qualitatively, the reaction profiles match those seen in Figure 11, with only small quantitative differences (Table 1). The mean unsigned deviation for barrier heights tends to increase slightly with larger basis sets, while decreasing for Δ*H* values for minima. This is indicative of the slow convergence of CASPT2 relative energies with respect to basis set size, while DFT is known to converge more quickly. We must raise a note of caution here: while we expect the XMS-CASPT2 data for the potential energy curves to be highly reliable (since we are considering the near-degeneracy of the lowest two singlet states), the enthalpy profiles may be misleading. In a work by Valsson, Filippi, and Casida, they demonstrated that DFT can sometimes be correct where CASPT2 has predicted a different phenomenon.^46^

**Table 1:** Mean Signed Deviations (MSD), Mean Unsigned Deviations (MUD), and Maximum Deviations for DFT Methods in Comparison to the XMS-CASPT2 Δ*H* Values for Transition States, and for Minima (Intermediates and Products), Relative to the Reactants^a^

	B3LYP			M11			ωB97X-D3		
	DZP	TZP	QZP	DZP	TZP	QZP	DZP	TZP	QZP
MSD (transition states)	4.2	6.1	7.3	5.8	8.5	10.0	7.7	9.1	10.4
MUD (transition states)	7.5	7.4	8.2	11.0	11.0	11.9	10.4	10.7	11.8
Max. deviation (transition states)	14.8	16.0	16.7	20.6	22.3	23.1	21.4	22.3	23.1
MSD (minima)	0.6	2.1	2.8	–4.3	–1.6	–0.5	–1.2	–0.2	0.6
MUD (minima)	6.4	5.5	5.5	14.3	12.6	12.4	11.8	10.1	10.2
Max. deviation (minima)	12.9	13.4	16.1	20.6	20.3	23.0	19.1	18.5	21.1

a All values are in kcal mol^–1^. DZP = 6-31G(d); TZP = Def2-TZVPP; QZP = Def2-QZVPP.

Given in Tables S1–S4 are the CI coefficients and % contribution of the two determinants corresponding to occupation of the two degenerate π* orbitals to the CASSCF wave function. For each of the reactions, the weight of the first determinant is always higher than the second, demonstrating that the degeneracy has been broken. The transition states for reactions (b, c and d) have very high contributions for one determinant, suggesting that these are well described by a single determinant. For reaction (a), the contribution of the first determinant rises from ∼48% of the CASSCF wave function at the first transition state to ∼93% at the second intermediate. Reaction (c) shows a range of ∼56 to ∼62% from the first transition state to the second transition state, although the product has a contribution of ∼97%. For the DFT calculated transition states, stability analysis does show a broken-symmetry (i.e., UKS) solution in some cases (reaction (a), first two transition states, (b,c)); in the current work, we effectively treat the RKS description of the transition state as a special case of the maximum overlap method (MOM),^47^ where we retain the spin symmetry by choice and where the comparison with the XMS-CASPT2 results above justify this.

### 4.2 Further Discussion

The analysis of the two lowest singlet electronic states and the triplet state for the first transition state of each reaction (i.e., when singlet oxygen first interacts with the organic molecule) given in Figures 4–7 and S1–S8 shows agreement with the idea postulated by Mullinax et al.^16^ that the singlet and triplet curves must cross at (or near) the transition state. These data also show that, at the transition state, the degeneracy of the two π* orbitals is lifted. This is further supported by analysis of the CASSCF orbitals and KS orbitals at the transition state for each of the reactions (Figures S11–S14), demonstrating significant energy differences between the two orbitals when interacting with the organic molecules. This lifting of degeneracy enables the use of single-reference spin-restricted approaches, leading to enthalpy changes that are broadly in agreement with CASPT2, especially the B3LYP functional. The different functionals examined do perform differently when considering quantitative agreement with CASPT2, although each of the KS methods is able to qualitatively describe each of the critical points of the reaction scheme, with the exception, of course, of isolated ^1^Δ_g_ O_2_.

## 5 Conclusions

We have examined the performance of spin-restricted KS DFT when applied to reactions of singlet oxygen with organic molecules for four prototypical reactions: 1,4-cycloaddition, the “ene” reaction, 1,2-cycloaddition, and a double hydrogen abstraction reaction. While KS DFT is known to be unsuitable for the correct description of isolated ^1^Δ_g_ O_2_, we have demonstrated that the degeneracy of the wave function is lifted once O_2_ reacts with another molecule since the (degenerate) π* orbitals interact nonsymmetrically, thus breaking the degeneracy. This is supported by calculations of the PES around the transition state geometry at the XMS-CASPT2 level, along with the CASSCF orbitals, where the two singlet states become significantly nondegenerate. The KS DFT approaches also show clear nondegeneracy of the singlet states and orbitals at this transition state. The calculated reaction profiles with the B3LYP approach agree well with the XMS-CASPT2 data, assuming that an appropriate correction is used for the singlet–triplet splitting of isolated O_2_. We recommend using the (spin-restricted) B3LYP approach when considering studying singlet oxygen reactions for the efficient and accurate computation of thermodynamic parameters but also to describe the electronic structure of the bulk of the molecular species without spin contamination.

## Supporting Information Available

Details of the active space selection, along with potential energy curves from the M11 and ωB97X-D3 reaction pathways; additional images of orbitals; CASSCF CI coefficients; the xyz geometries for the reaction pathways calculated using DFT; and the three different basis sets (PDF)
XYZ geometries of stationary points (ZIP)
