# Supplementary material for: Spin-Restricted Descriptions of Singlet Oxygen Reactions from XMS-CASPT2 Benchmarks
Source: J Phys Chem A. 2024 May 13;128(20):4128–37. doi: 10.1021/acs.jpca.4c00744 (PMC11129307; doi:10.1021/acs.jpca.4c00744)
Supplement: Supplementary file 1 — jp4c00744_si_001.pdf [file jp4c00744_si_001.pdf]

SUPPORTING INFORMATION

Spin-Restricted Descriptions of Singlet Oxygen Reactions from XMS-CASPT2 Benchmarks

Max Winslow,[1,2] *Alexander Hazelby,*[1] *David Robinson*[1,*].

[1]Department of Chemistry and Forensics, School of Science and Technology, Nottingham Trent University, Clifton Lane, Nottingham, NG11 8NS, United Kingdom.

[2]Current address: School of Chemistry, University of Nottingham, University Park, Nottingham, NG7 2RD, United Kingdom.

SUPPORTING INFORMATION

CASSCF Active Space Selection

Reaction (a)

The two π and two π* orbitals from $^1O_2$ and two π and two π* orbitals from butadiene were included, giving an active space of (10,8).

Reaction (b)

The two π and two π* orbitals from $^1O_2$ were included, along with the π and two π* orbitals and one of the C-H σ (and σ*) orbitals from butadiene, giving an active space of (10,8). The C-H bond was the one pointing towards the O_2 molecule (from the $-CH_3$ group).

Reaction (c)

The two π and two π* orbitals from $^1O_2$ and the π and π* orbitals from ethene were included, giving an active space of (8,6).

Reaction (d)

The initial orbitals were taken from the products (1,2-dimethoxyethene and hydrogen peroxide), as these were more well defined. The π and π* orbitals of 1,2-dimethoxyethene were included, along with the full valence space of orbitals from hydrogen peroxide. This gave an active space of (16,12).

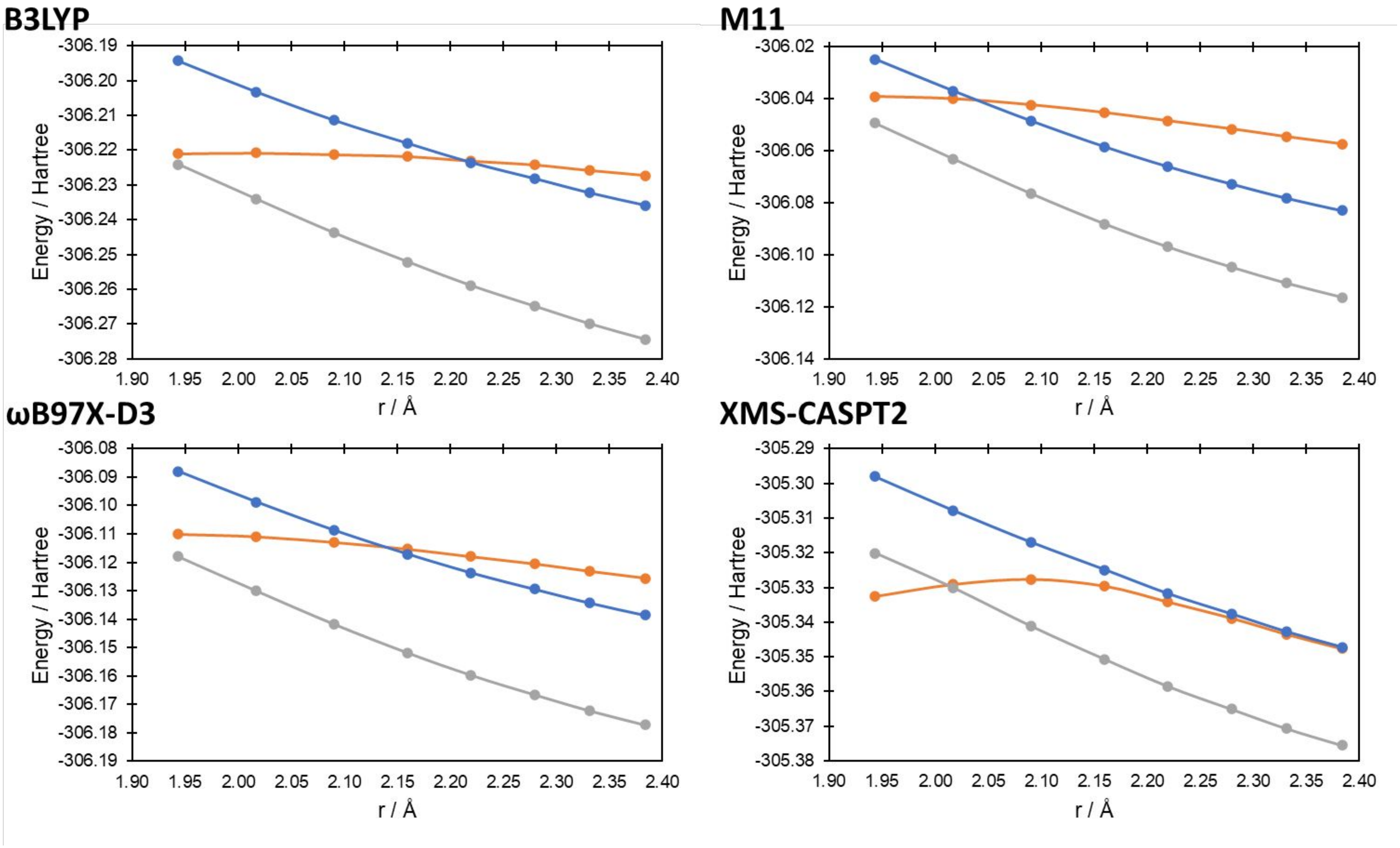

Figure S1. Potential energy curves for the lowest triplet and two lowest singlet energy states at the first transition state geometry (r_{C-O} = 1.94 Å; data point on the left) and along the intrinsic reaction coordinate calculated at the M11/6-31G(d) level for reaction (a). The distance on the x-axis corresponds to the C-O bond distance as the bond forms (second transition state shown in Figure 2a of the main text; M11 does not find the first transition state) along the reaction coordinate. The triplet energies are the gray lines, while the singlet energies are in orange and blue.

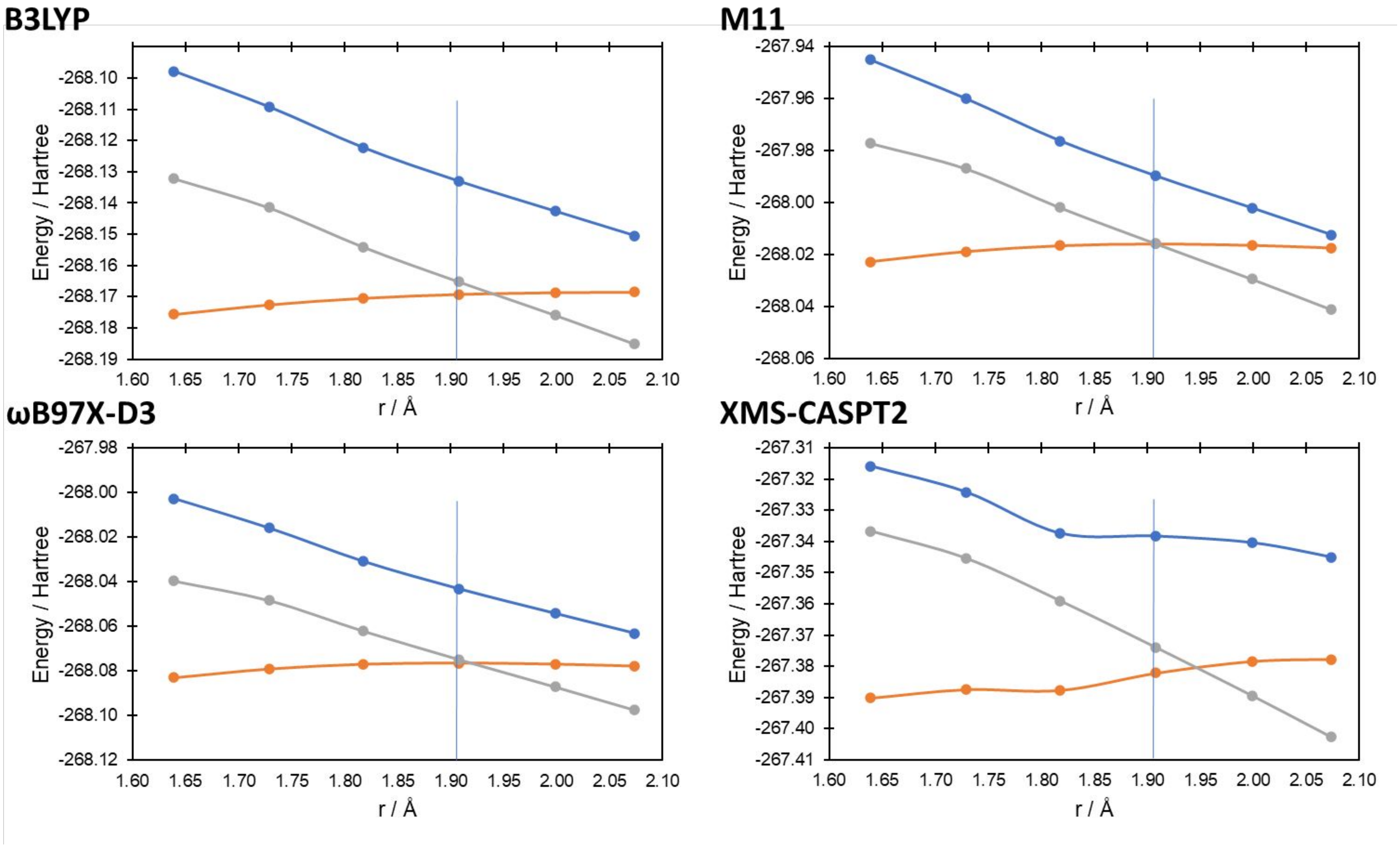

Figure S2. Potential energy curves for the lowest triplet and two lowest singlet energy states at the first transition state geometry ($r_{C\text{-}O}$ = 1.91 Å; data point highlighted with a vertical line) and along the intrinsic reaction coordinate calculated at the M11/6-31G(d) level for reaction (b). The distance on the x-axis corresponds to the C-O bond distance as the bond forms (see Figure 2b) along the reaction coordinate. The triplet energies are the gray lines, while the singlet energies are in orange and blue.

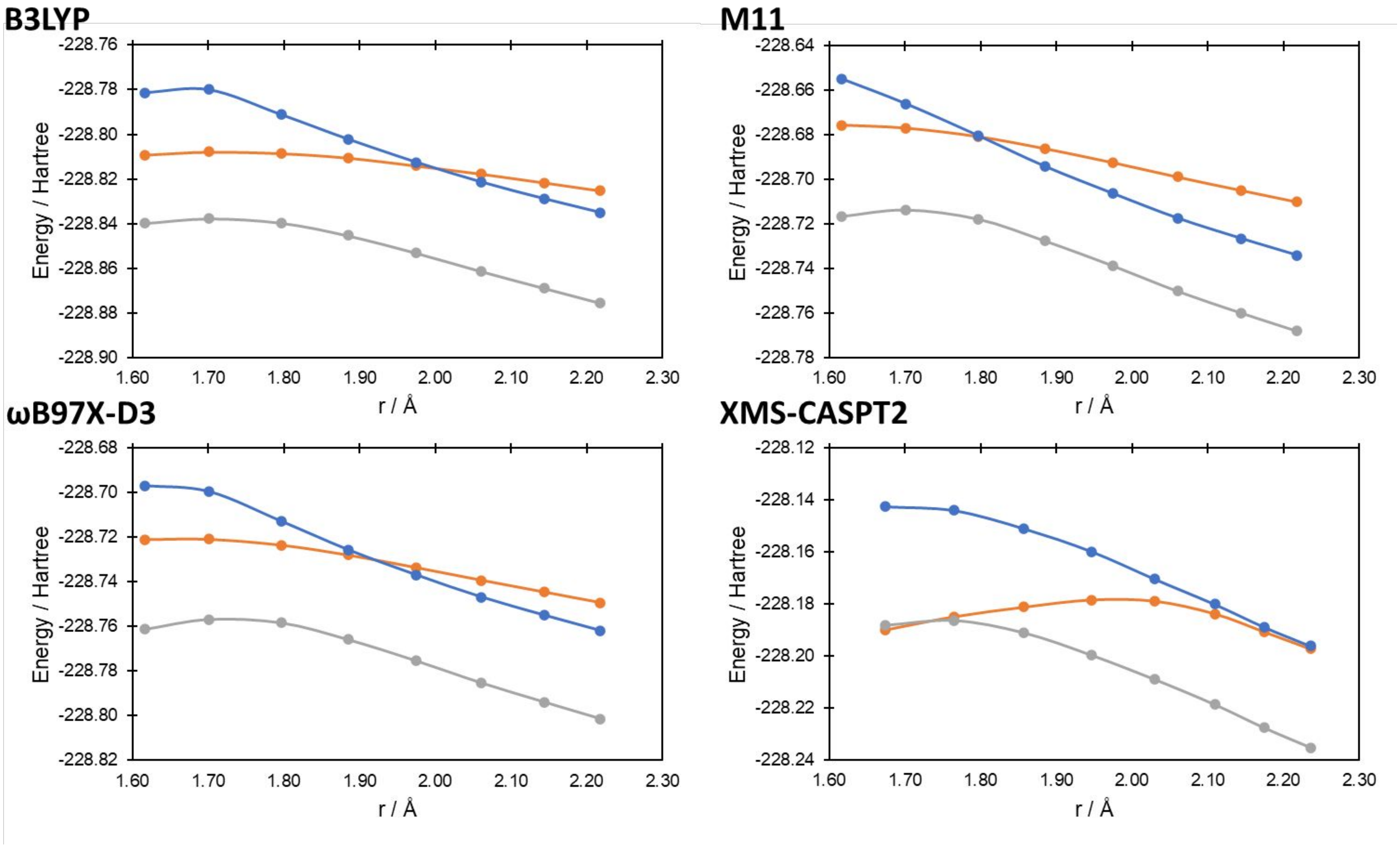

Figure S3. Potential energy curves for the lowest triplet and two lowest singlet energy states at the first transition state geometry ($r_{C\text{-}O}$ = 1.62 Å; data point on the left) and along the intrinsic reaction coordinate calculated at the M11/6-31G(d) level for reaction (c). The distance on the x-axis corresponds to the C-O bond distance as the bond forms (see Figure 2c) along the reaction coordinate. The triplet energies are the gray lines, while the singlet energies are in orange and blue.

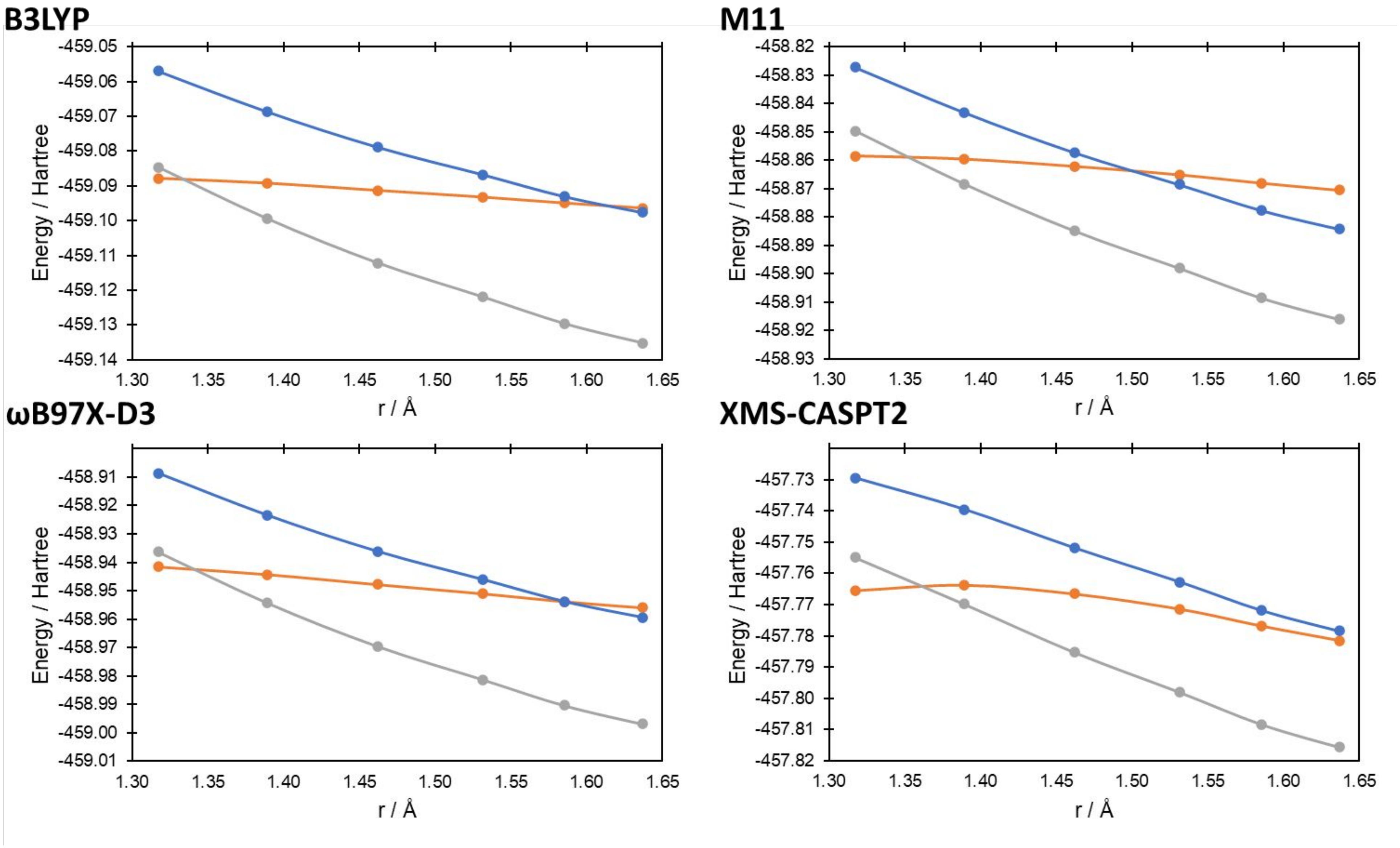

Figure S4. Potential energy curves for the lowest triplet and two lowest singlet energy states at the first transition state geometry ($r_{O\text{-}H}$ = 1.32 Å; data point on the left) and along the intrinsic reaction coordinate calculated at the M11/6-31G(d) level for reaction (d). The distance on the x-axis corresponds to the O-H bond distance as the bond forms (see Figure 2d) along the reaction coordinate. The triplet energies are the gray lines, while the singlet energies are in orange and blue.

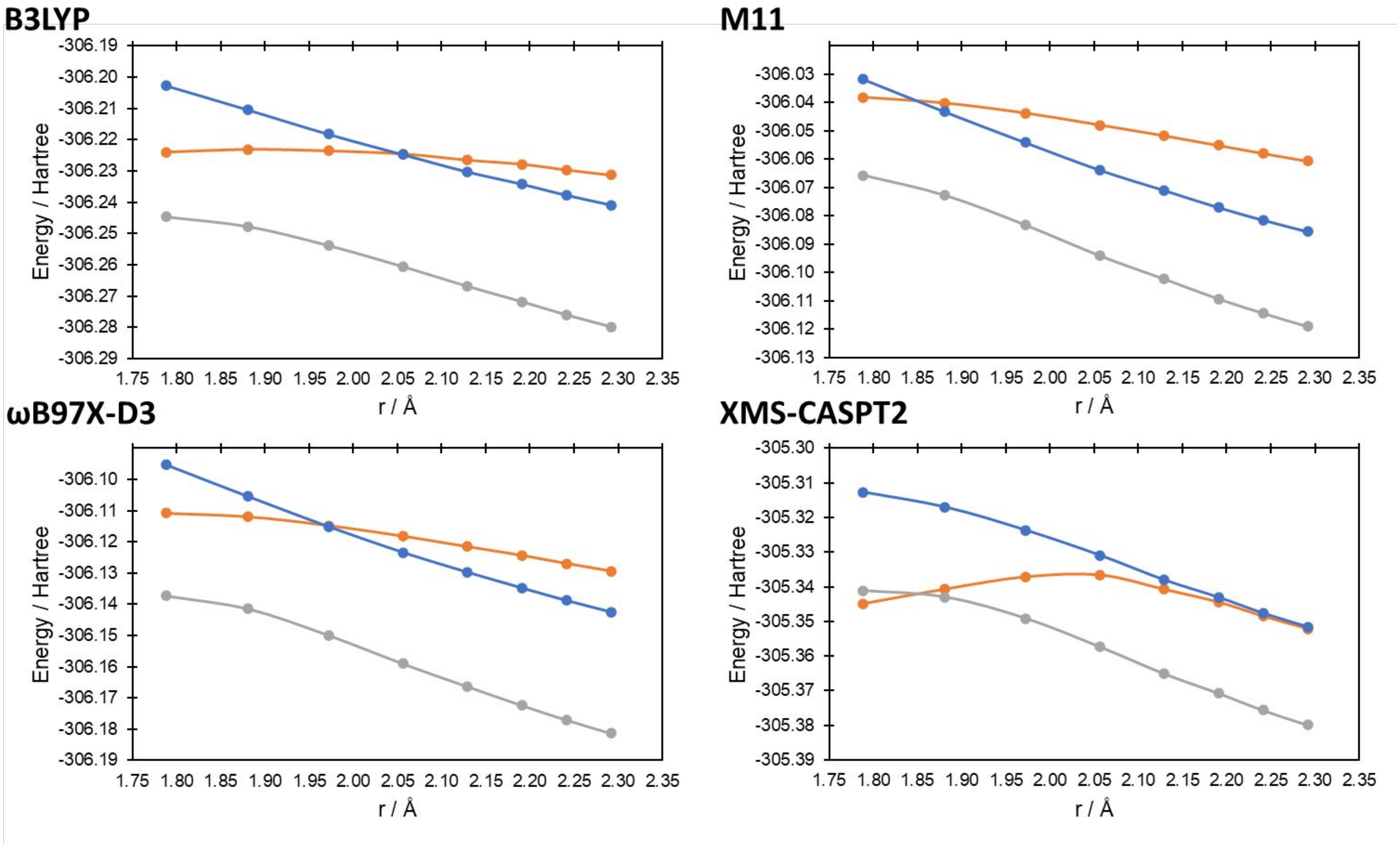

Figure S5. Potential energy curves for the lowest triplet and two lowest singlet energy states at the first transition state geometry ($r_{C\text{-}O}$ = 1.79 Å; data point on the left) and along the intrinsic reaction coordinate calculated at the ωB97X-D3/6-31G(d) level for reaction (a). The distance on the x-axis corresponds to the C-O bond distance as the bond forms (see Figure 2a of the main text) along the reaction coordinate. The triplet energies are the gray lines, while the singlet energies are in orange and blue.

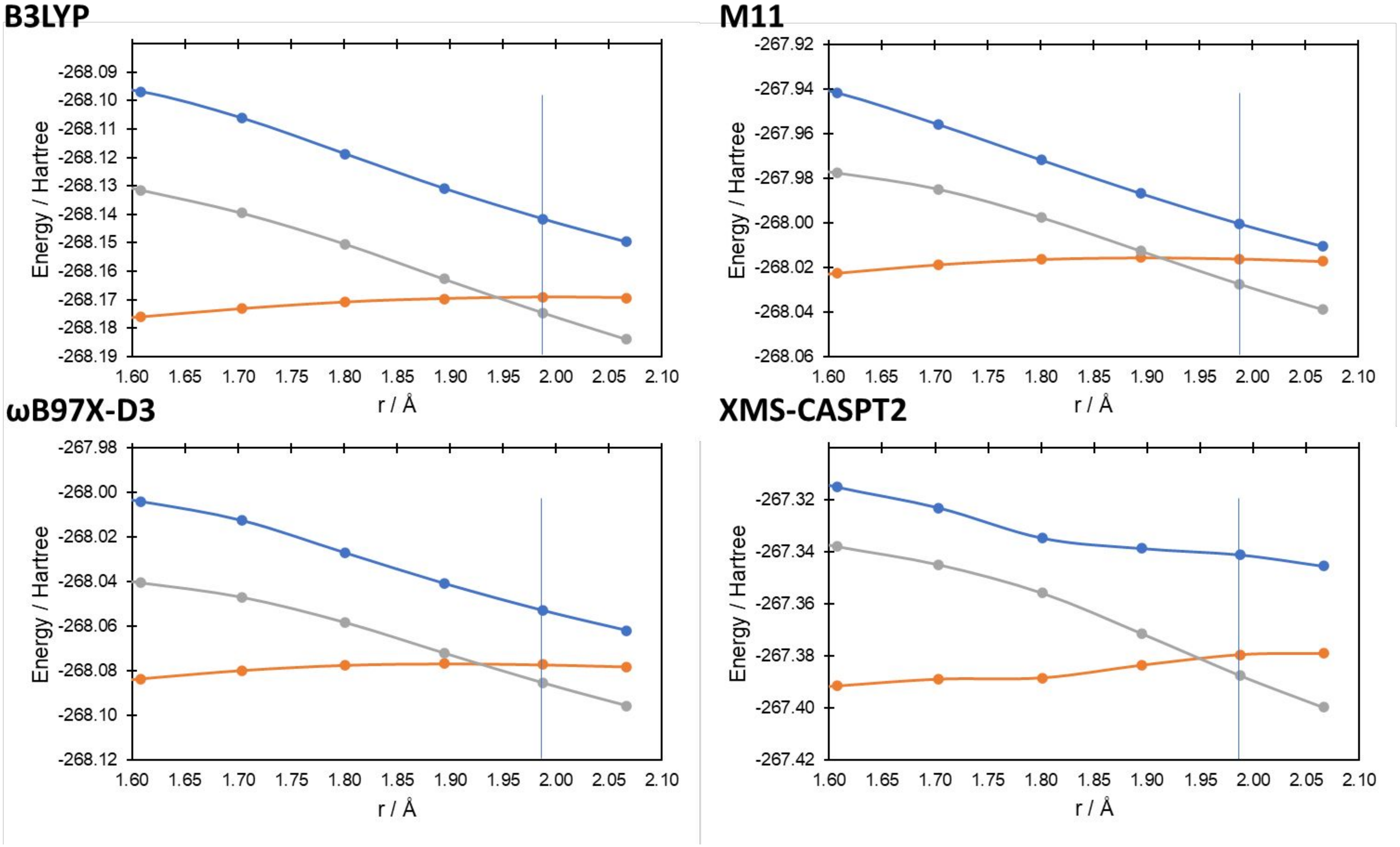

Figure S6. Potential energy curves for the lowest triplet and two lowest singlet energy states at the first transition state geometry ($r_{C\text{-}O}$ = 1.89 Å; data point highlighted with a vertical line) and along the intrinsic reaction coordinate calculated at the ωB97X-D3/6-31G(d) level for reaction (b). The distance on the x-axis corresponds to the C-O bond distance as the bond forms (see Figure 2b) along the reaction coordinate. The triplet energies are the gray lines, while the singlet energies are in orange and blue.

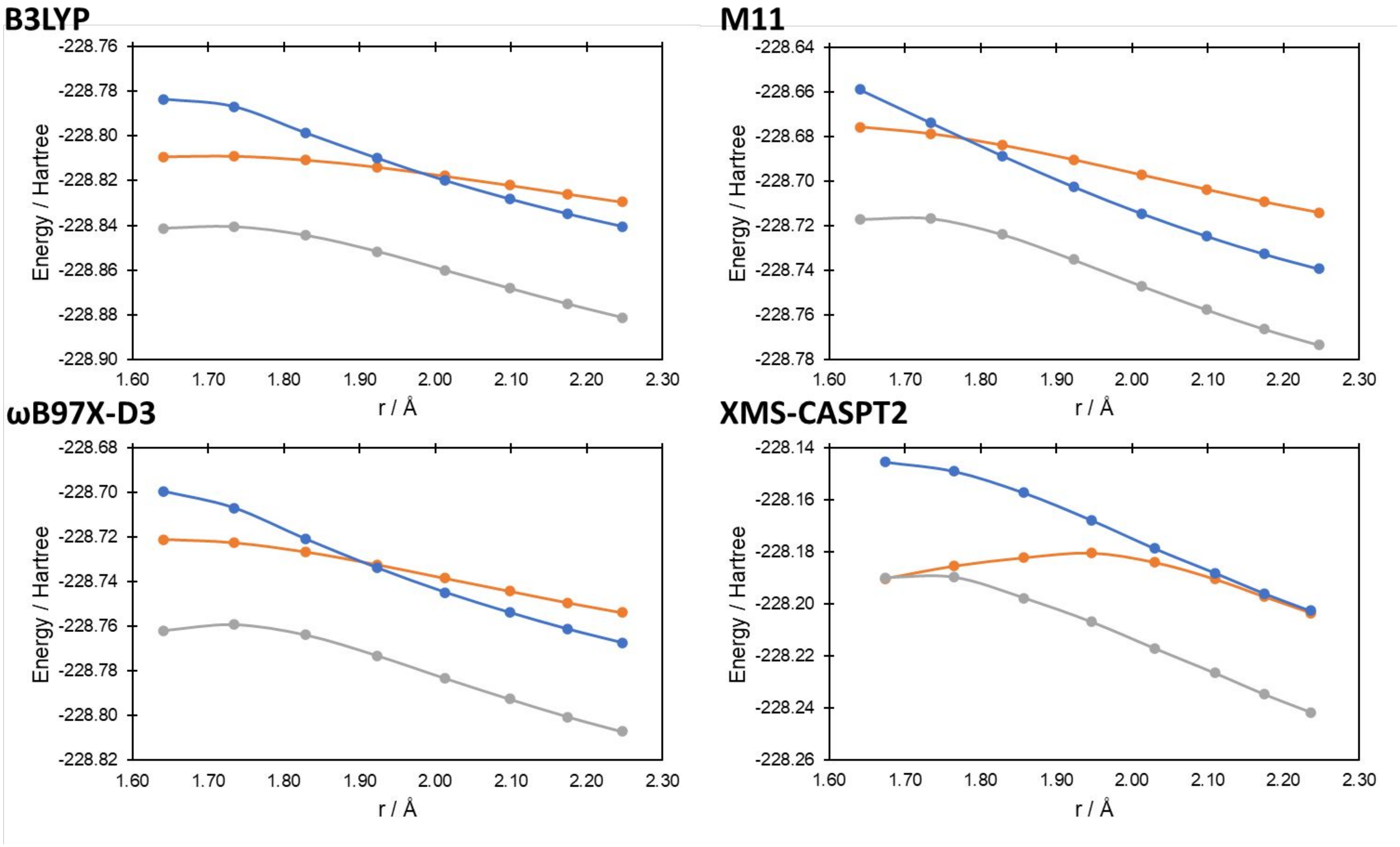

Figure S7. Potential energy curves for the lowest triplet and two lowest singlet energy states at the first transition state geometry ($r_{C\text{-}O}$ = 1.64 Å; data point on the left) and along the intrinsic reaction coordinate calculated at the ωB97X-D3/6-31G(d) level for reaction (c). The distance on the x-axis corresponds to the C-O bond distance as the bond forms (see Figure 2c) along the reaction coordinate. The triplet energies are the gray lines, while the singlet energies are in orange and blue.

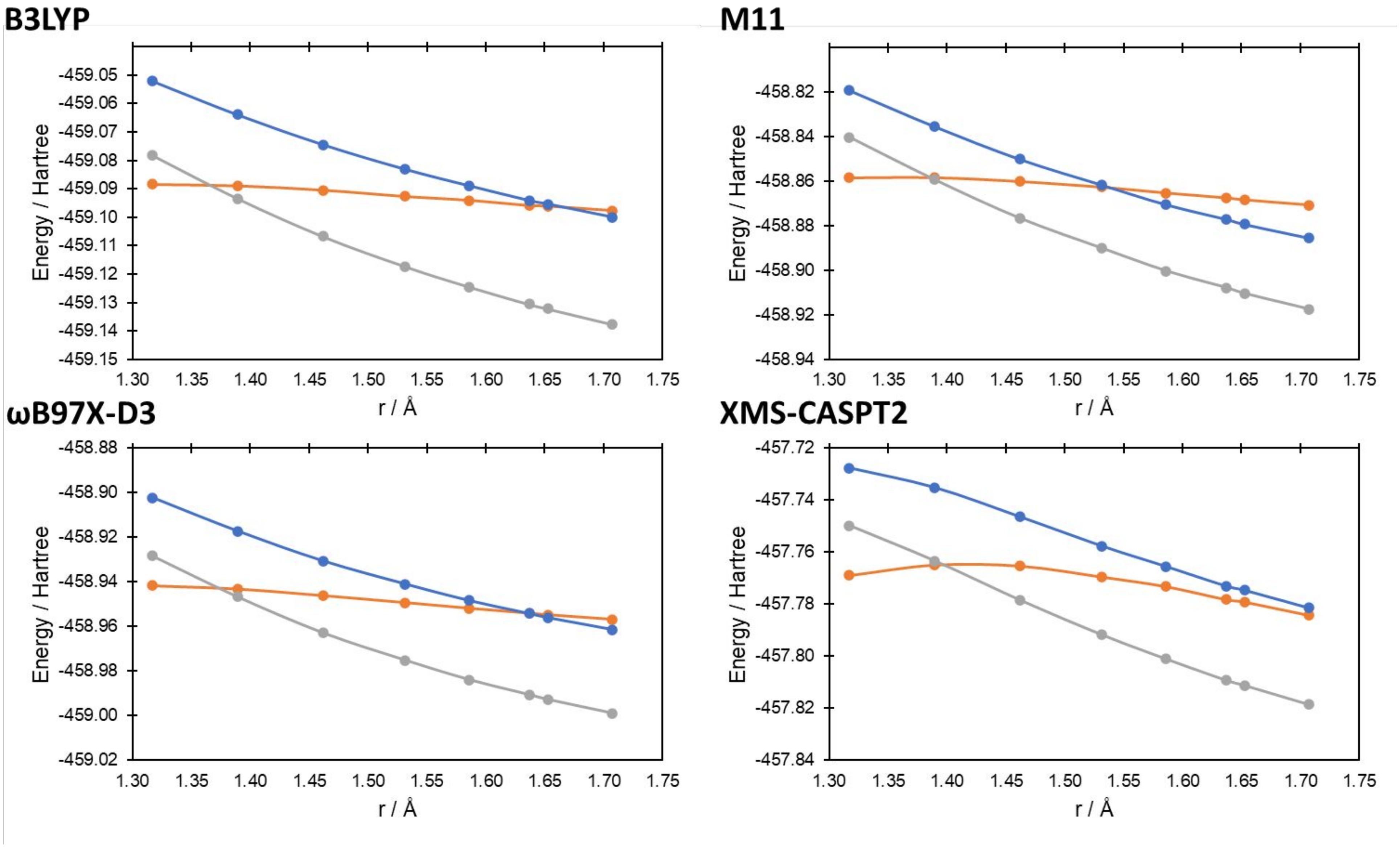

Figure S8. Potential energy curves for the lowest triplet and two lowest singlet energy states at the first transition state geometry ($r_{O\text{-}H}$ = 1.32 Å; data point on the left) and along the intrinsic reaction coordinate calculated at the ωB97X-D3/6-31G(d) level for reaction (d). The distance on the x-axis corresponds to the O-H bond distance as the bond forms (see Figure 2d) along the reaction coordinate. The triplet energies are the gray lines, while the singlet energies are in orange and blue.

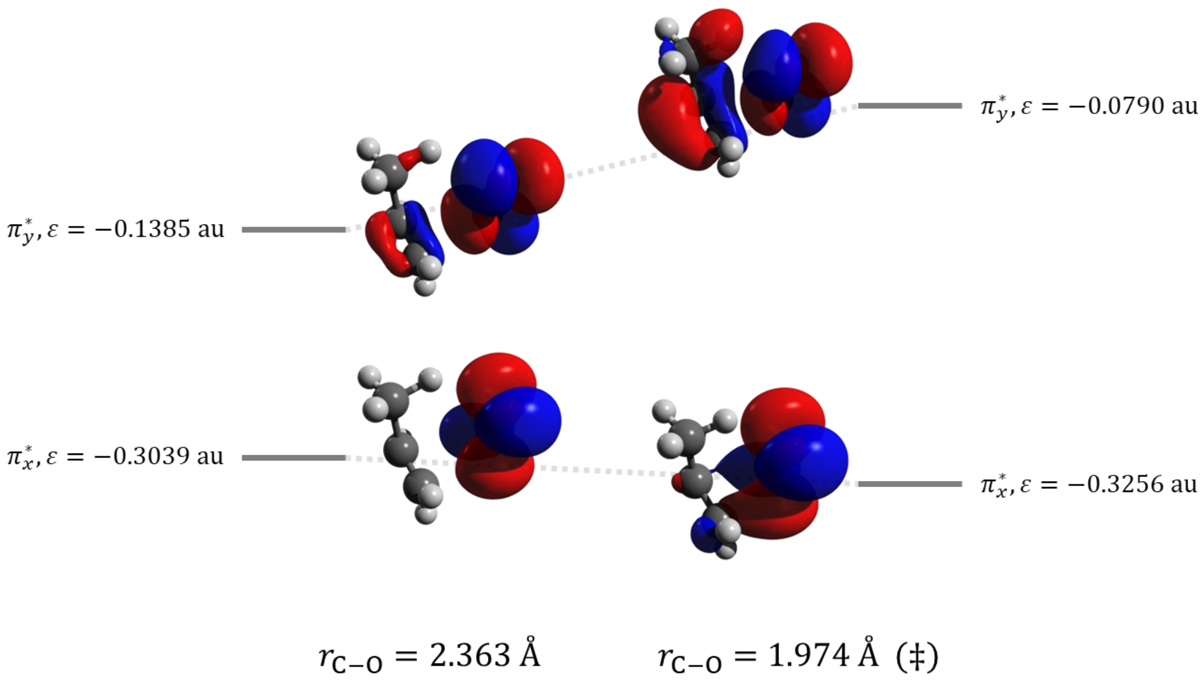

Figure S9. Schematic of the two π* orbitals of O_2 interacting with propene a short distance from the transition state (left), and at the transition state (right) for reaction (b). The orbitals come from CASSCF/6-31G(d) calculations.

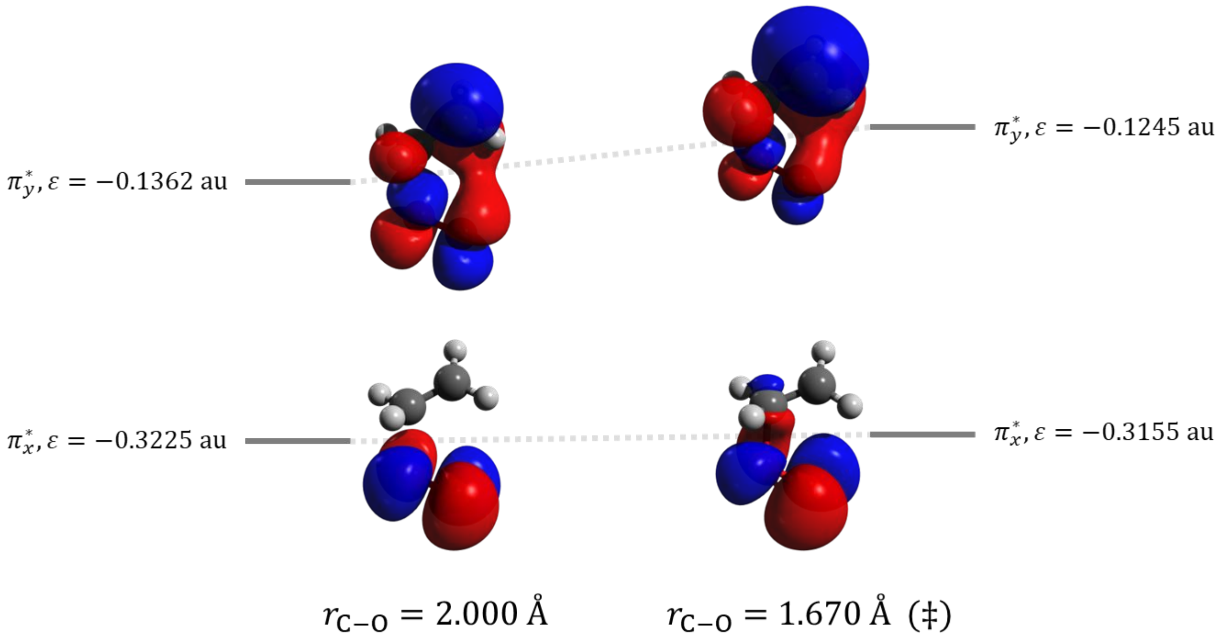

Figure S10. Schematic of the two π* orbitals of O_2 interacting with ethene a short distance from the transition state (left), and at the transition state (right) for reaction (c). The orbitals come from CASSCF/6-31G(d) calculations.

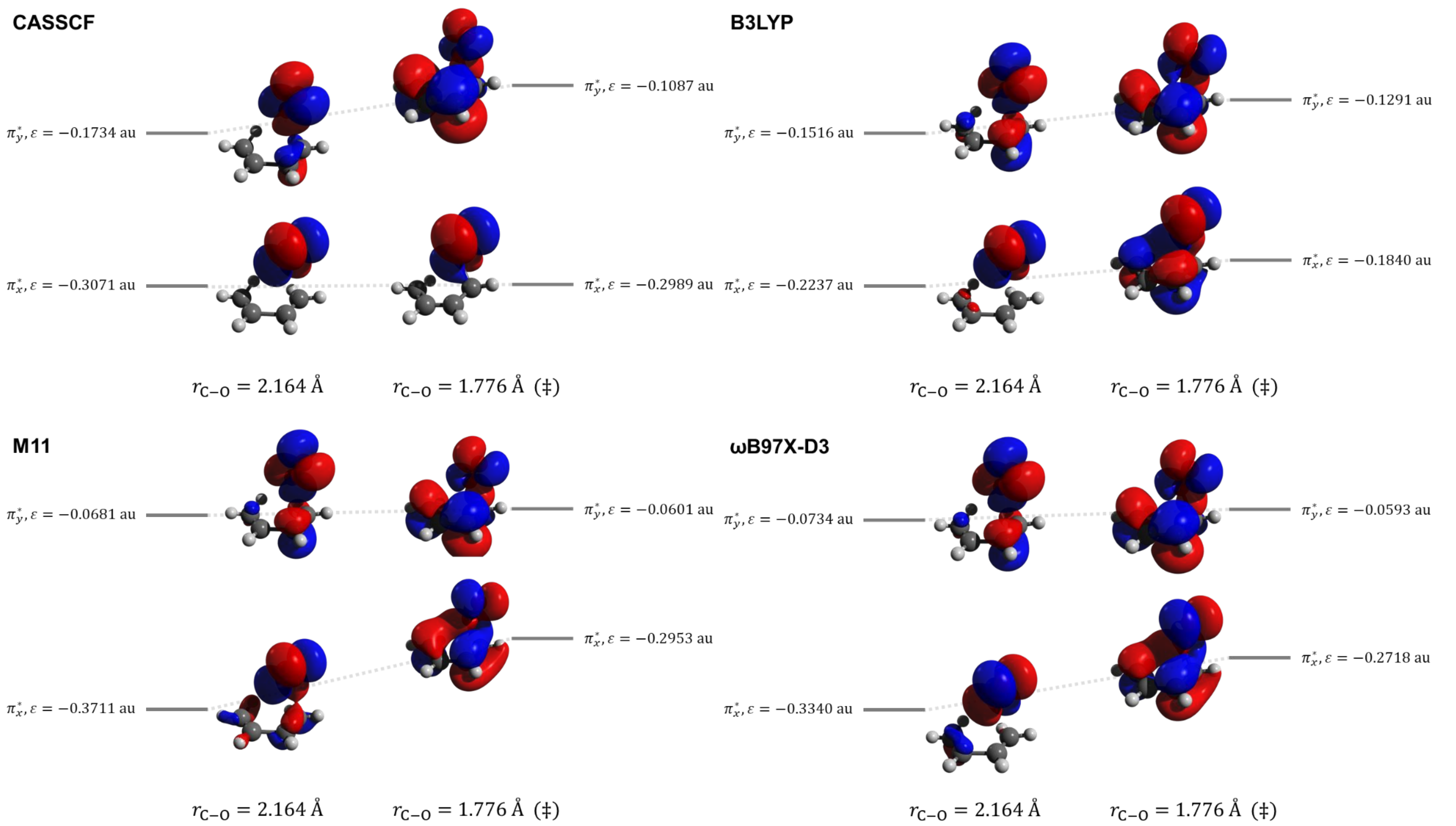

Figure S11. Schematic of the two π* orbitals of O_2 interacting with butadiene a short distance from the transition state (left for each method), and at the transition state (right for each method) for reaction (a).

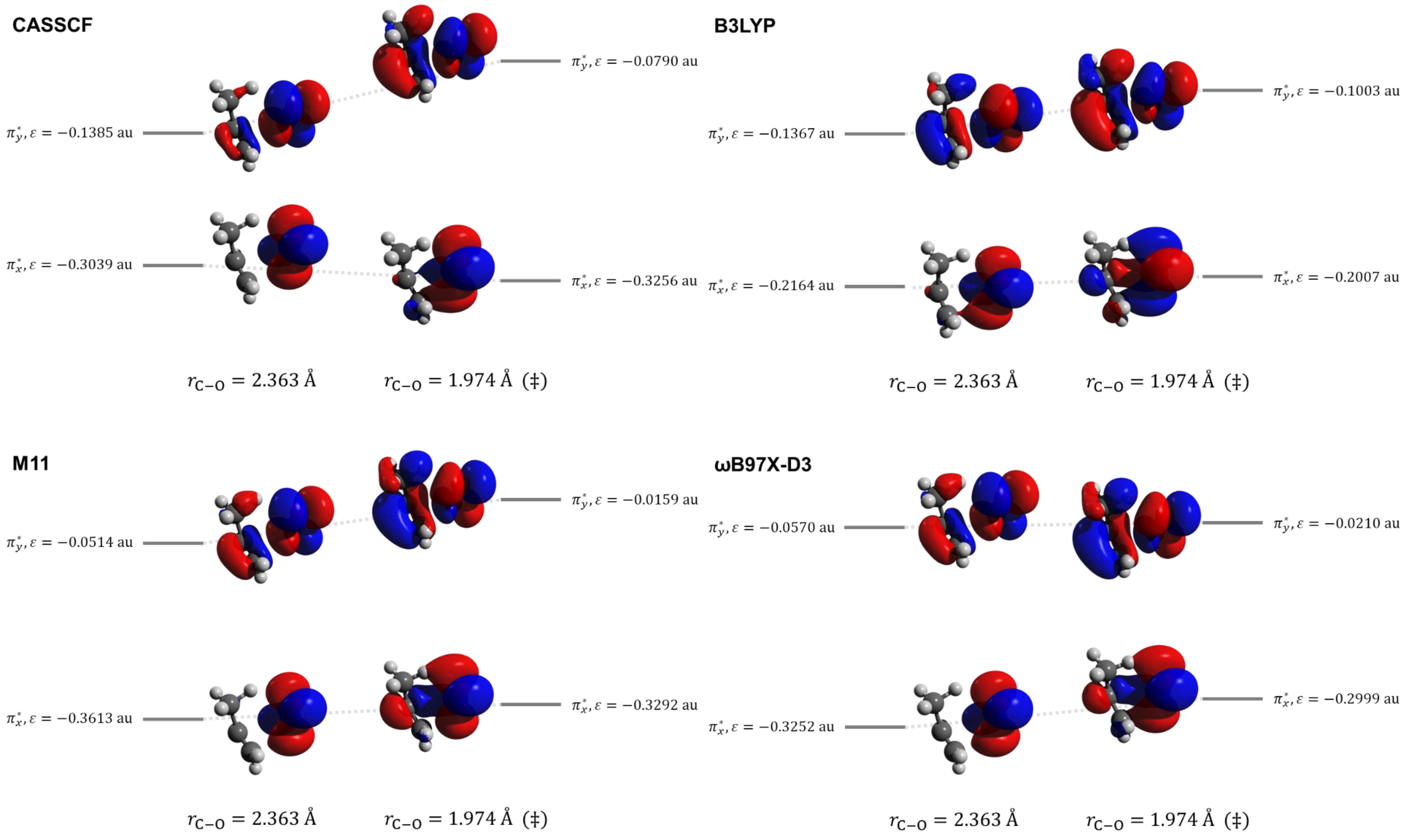

Figure S12. Schematic of the two π* orbitals of O_2 interacting with propene a short distance from the transition state (left for each method), and at the transition state (right for each method) for reaction (b).

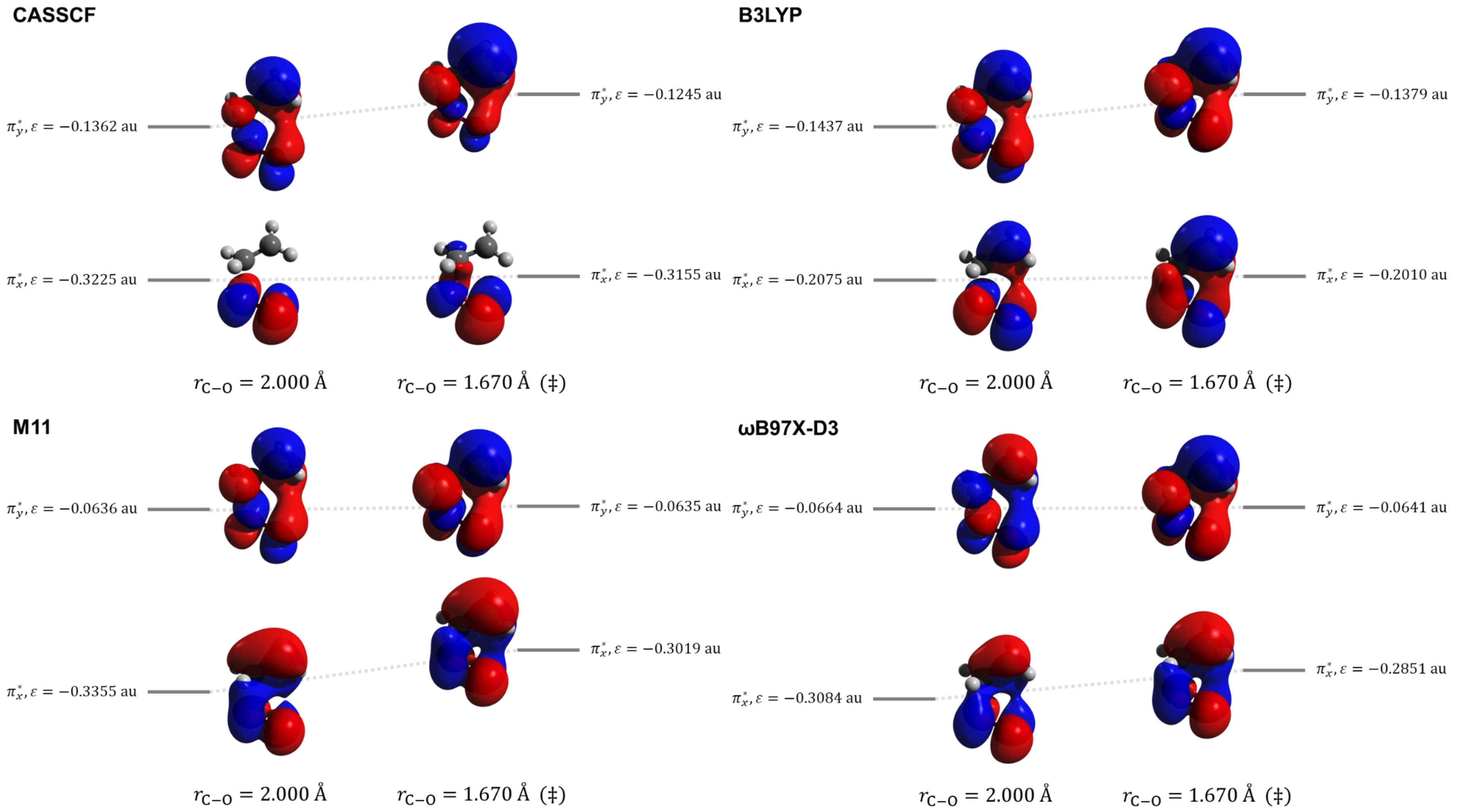

Figure S13. Schematic of the two π* orbitals of O_2 interacting with ethene a short distance from the transition state (left for each method), and at the transition state (right for each method) for reaction (c).

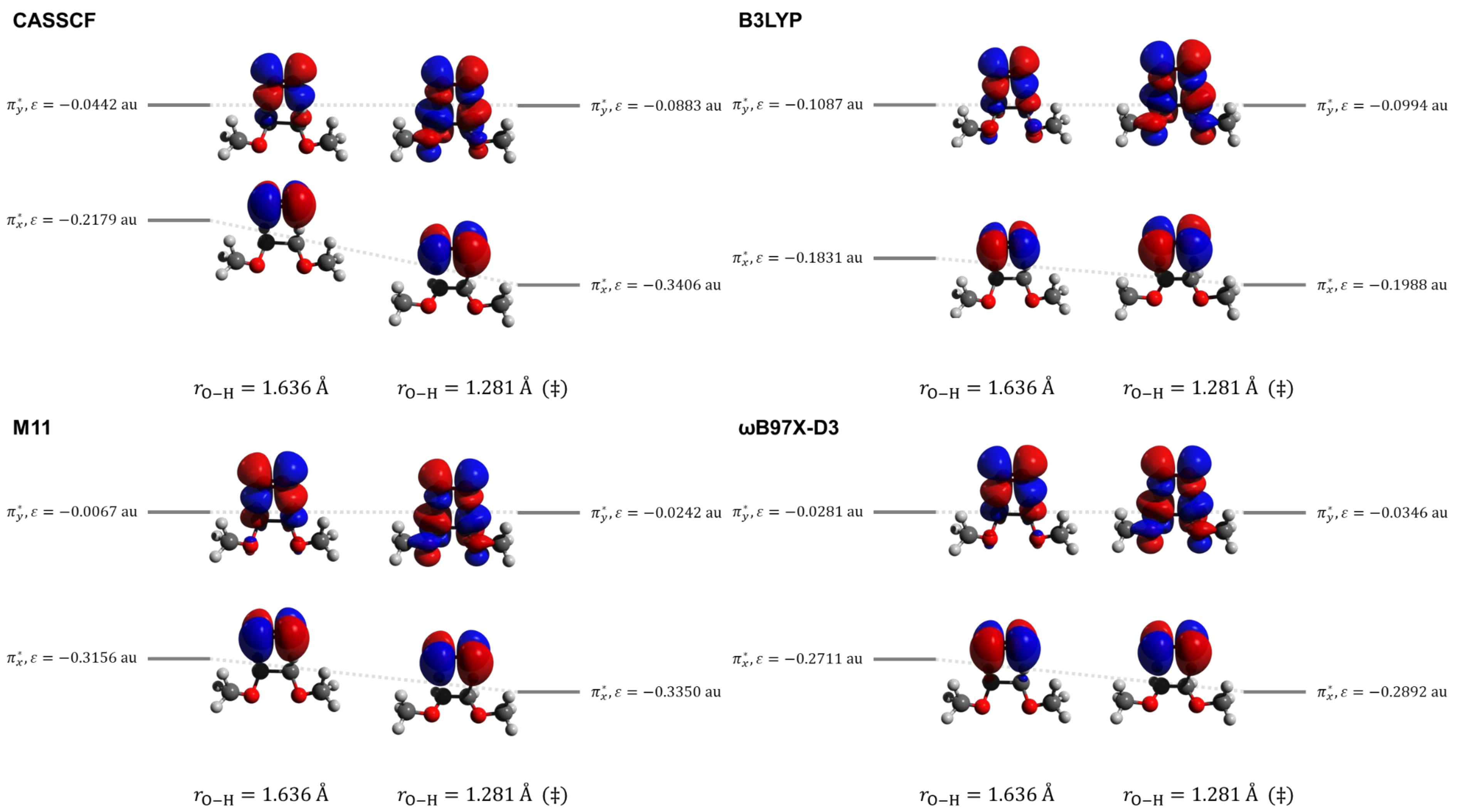

Figure S14. Schematic of the two π* orbitals of O_2 interacting with 1,2-dimethoxyethane a short distance from the transition state (left for each method), and at the transition state (right for each method) for reaction (d).

Reaction (a)

TS1

Determinant	CI coeff	% weight
22222...	-0.692170	47.91%
2222.2..	0.627934	39.43%

Intermediate 1

Determinant	CI coeff	% weight
22222...	-0.791058	62.58%
2222.2..	0.512360	26.25%

TS2

Determinant	CI coeff	% weight
22222...	0.793794	63.01%
2222.2..	-0.516630	26.69%

Intermediate 2

Determinant	CI coeff	% weight
22222...	-0.965431	93.21%
2222.2..	0.201436	4.06%

TS3

Determinant	CI coeff	% weight
22222...	0.946361	89.56%
2222.2..	-0.193911	3.76%

Product

Determinant	CI coeff	% weight
22222...	0.968880	93.87%
2222.2..	-0.170114	2.89%

Table S1. CASSCF CI coefficients and % weight of the two determinants in the wavefunction of the ground state, corresponding to occupation of either of the two degenerate π^* orbitals (and their successors) for each of the stationary points of reaction (a).

Reaction (b)

TS

Determinant	CI coeff	% weight
22222...	0.909242	82.67
2222.2..	-0.267168	7.14

Product

Determinant	CI coeff	% weight
22222...	-0.956611	91.51
2222.2..	0.196675	3.87

Table S2. CASSCF CI coefficients and % weight of the two determinants in the wavefunction of the ground state, corresponding to occupation of either of the two degenerate π^* orbitals (and their successors) for each of the stationary points of reaction (b).

Reaction (c)

TS1

Determinant	CI coeff	% weight
2222..	0.745938	55.64%
222.2.	-0.637140	40.59%

Int

Determinant	CI coeff	% weight
2222..	-0.768554	59.07%
222.2.	0.603508	36.42%

TS2

Determinant	CI coeff	% weight
2222..	-0.787028	61.94%
222.2.	0.576933	33.29%

Product

Determinant	CI coeff	% weight
2222..	-0.986173	97.25%
222.2.	0.063425	0.40%

Table S3. CASSCF CI coefficients and % weight of the two determinants in the wavefunction of the ground state, corresponding to occupation of either of the two degenerate π^* orbitals (and their successors) for each of the stationary points of reaction (c).

Reaction (d)

TS

**Determinant**	**CI coeff**	**% weight**
22222222....	-0.940093	88.38%
2222222.2...	0.194300	3.78%

H_2O_2

**Determinant**	**CI coeff**	**% weight**
2222222...	-0.969774	94.05%
222222.2..	0.168918	2.85%

Product

**Determinant**	**CI coeff**	**% weight**
2.	0.979049	95.85%
.2	-0.203623	4.15%

Table S4. CASSCF CI coefficients and % weight of the two determinants in the wavefunction of the ground state, corresponding to occupation of either of the two degenerate π^* orbitals (and their successors) for each of the stationary points of reaction (d).

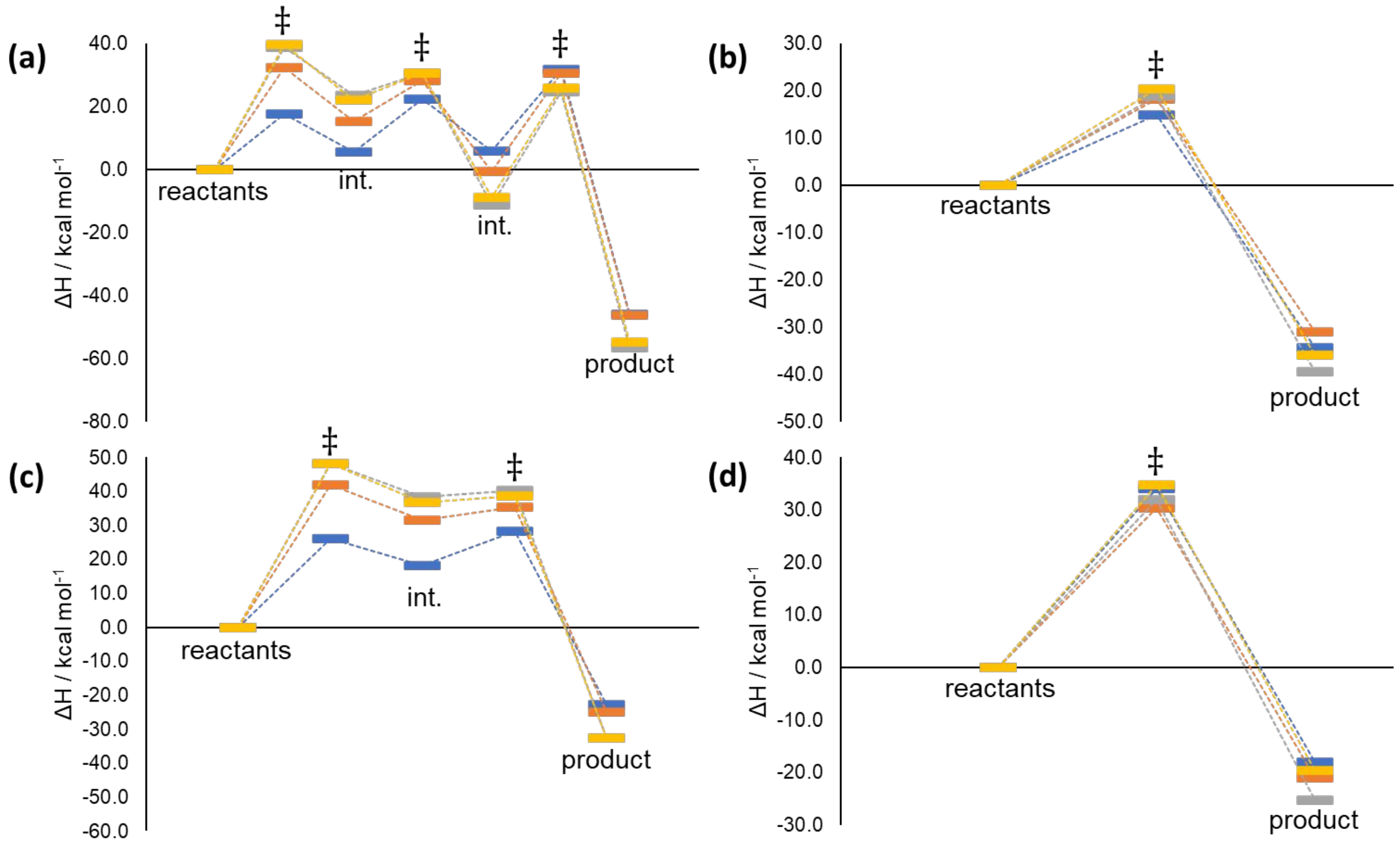

Figure S15. Reaction enthalpy profiles for reactions (a), (b), (c) and (d) using the def2-TZVPP basis set. The absolute value of the singlet oxygen energy for the DFT methods is taken as the sum of the triplet ground state energy plus the triplet-singlet gap taken from the XMS-CASPT2 calculations. Key: XMS-CASPT2 (blue), B3LYP (orange), M11 (grey), ωB97X-D3 (yellow).

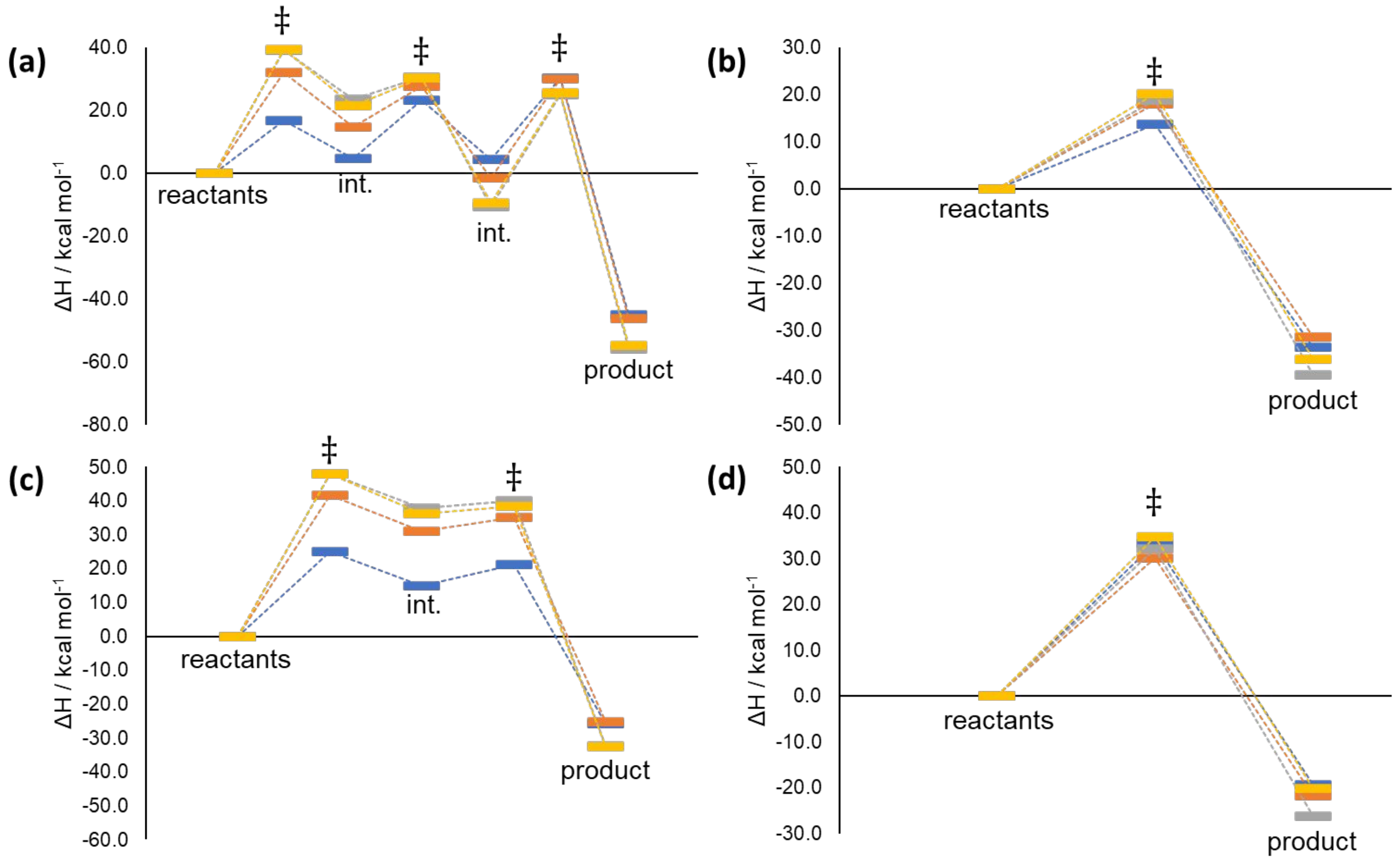

Figure S16. Reaction enthalpy profiles for reactions (a), (b), (c) and (d) using the def2-QZVPP basis set. The absolute value of the singlet oxygen energy for the DFT methods is taken as the sum of the triplet ground state energy plus the triplet-singlet gap taken from the XMS-CASPT2 calculations. Key: XMS-CASPT2 (blue), B3LYP (orange), M11 (grey), ωB97X-D3 (yellow).

Basis set	ΔE_{S-T} / kcal mol^{-1}			
	XMS-CASPT2	MSM-B3LYP	MSM-M11	MSM-ωB97X-D3
6-31G(d)	24.77	20.90	21.67	23.84
def2-TZVPP	23.77	20.18	19.97	23.29
def2-QZVPP	23.46	20.01	20.37	23.14

Table S5. Values of the singlet-triplet gap between the $^3\Sigma_g^-$ and $a^1\Delta_g$ states calculated using XMS-CASPT2, multiplet-sum method (MSM) variants of B3LYP, M11 and ωB97X-D3. The best estimate of the gap is 22.65 kcal mol^{-1} (Theor. Chem. Acc. **2021**, *140*, 154).
